# Complete chloroplast genomes and phylogeny in three *Euterpe* palms (*E*. *edulis*, *E*. *oleracea* and *E*. *precatoria*) from different Brazilian biomes

**DOI:** 10.1371/journal.pone.0266304

**Published:** 2022-07-28

**Authors:** Ana Flávia Francisconi, Luiz Augusto Cauz-Santos, Jonathan Andre Morales Marroquín, Cássio van den Berg, Alessandro Alves-Pereira, Luciano Delmondes de Alencar, Doriane Picanço-Rodrigues, Cesar Augusto Zanello, Marcones Ferreira Costa, Maria Teresa Gomes Lopes, Elizabeth Ann Veasey, Maria Imaculada Zucchi

**Affiliations:** 1 Programa de Pós-Graduação em Genética e Biologia Molecular, Universidade Estadual de Campinas, Campinas, São Paulo, Brasil; 2 Department of Botany and Biodiversity Research, University of Vienna, Wien, Austria; 3 Departamento de Ciências Biológicas, Universidade Estadual de Feira de Santana, Feira de Santana, Bahia, Brasil; 4 Departamento de Genética, Universidade de São Paulo, Piracicaba, São Paulo, Brasil; 5 Departamento de Biologia Vegetal, Universidade Estadual de Campinas, Campinas, São Paulo, Brasil; 6 Departamento de Biologia, Universidade Federal do Amazonas, Manaus, Amazonas, Brasil; 7 Campus Amílcar Ferreira Sobral, Universidade Federal do Piauí, Floriano, Piauí, Brasil; 8 Departamento de Produção Animal e Vegetal, Universidade Federal do Amazonas, Manaus, Amazonas, Brasil; 9 Agência Paulista de Tecnologia dos Agronegócios, Piracicaba, São Paulo, Brasil; Central University of Punjab, INDIA

## Abstract

The Brazilian palm fruits and hearts-of-palm of *Euterpe edulis*, *E*. *oleracea and E*. *precatoria* are an important source for agro-industrial production, due to overexploitation, conservation strategies are required to maintain genetic diversity. Chloroplast genomes have conserved sequences, which are useful to explore evolutionary questions. Besides the plastid DNA, genome skimming allows the identification of other genomic resources, such as single nucleotide polymorphisms (SNPs), providing information about the genetic diversity of species. We sequenced the chloroplast genome and identified gene content in the three *Euterpe* species. We performed comparative analyses, described the polymorphisms among the chloroplast genome sequences (repeats, indels and SNPs) and performed a phylogenomic inference based on 55 palm species chloroplast genomes. Finally, using the remaining data from genome skimming, the nuclear and mitochondrial reads, we identified SNPs and estimated the genetic diversity among these *Euterpe* species. The *Euterpe* chloroplast genomes varied from 159,232 to 159,275 bp and presented a conserved quadripartite structure with high synteny with other palms. In a pairwise comparison, we found a greater number of insertions/deletions (indels = 93 and 103) and SNPs (284 and 254) between *E*. *edulis*/*E*. *oleracea* and *E*. *edulis*/*E*. *precatoria* when compared to *E*. *oleracea*/*E*. *precatoria* (58 indels and 114 SNPs). Also, the phylogeny indicated a closer relationship between *E*. *oleracea*/*E*. *precatoria*. The nuclear and mitochondrial genome analyses identified 1,077 SNPs and high divergence among species (F_ST_ = 0.77), especially between *E*. *edulis* and *E*. *precatoria* (F_ST_ = 0.86). These results showed that, despite the few structural differences among the chloroplast genomes of these *Euterpe* palms, a differentiation between *E*. *edulis* and the other *Euterpe* species can be identified by point mutations. This study not only brings new knowledge about the evolution of *Euterpe* chloroplast genomes, but also these new resources open the way for future phylogenomic inferences and comparative analyses within Arecaceae.

## Introduction

The palm family (Arecaceae) comprises 188 genera and ca. 2,400 species, distributed throughout tropical and subtropical regions [1, 2]. Palms are often keystone species, providing ecosystem services, shaping their environment and offering many products used for fabrics, fuel, food, medicine and as ornamentals [2–4]. Within Arecaceae, the genus *Euterpe* originated in South America and includes seven species distributed throughout Central America and tropical South America [2, 5, 6]. In Brazil, among the native species of *Euterpe*, *E*. *edulis* Mart., *E*. *oleracea* Mart. and *E*. *precatoria* Mart. are the most important from an agro-industrial point of view [7].

*Euterpe edulis* is endemic to the Atlantic Forest and has substantial importance for the functioning of the ecosystem [8, 9]. However, the overexploitation of heart-of-palm [10, 11] has placed this palm species in the Brazilian Flora Red Book [12] as “vulnerable (VU)”, which makes conservation strategies for it extremely necessary. Recently, as an alternative to palm heart-of-palm extraction, local communities have started to cultivate this species for fruit pulp extraction, due to the similarity with the Amazonian açaí (*E*. *oleracea* and *E*. *precatoria*) [9, 10, 13]. This practice has been fundamental to capture and conserve the species genetic diversity present in older forests [10].

*Euterpe oleracea* and *E*. *precatoria* are the main sources for the production of açaí fruit pulp, estimated to be 760,000 tons in 2017, mostly in the northern region of Brazil (98,36% of the national production) [14, 15]. *E*. *oleracea* is a multiple-stemmed palm, commonly found in the Amazon estuary, especially prevalent in the Brazilian states of Pará, Maranhão and Amapá [15–17]. However, in the last 20 years, the intense demand for açaí fruit in Pará resulted in a significant loss of local tree species richness, which presumably is occurring after decades of thinning to reduce interspecific competition with açai palm trees [18]. This resulted in a reduction of pollinators, which in turn decreased the fruit production [19]. *E*. *precatoria* is a single stemmed palm, predominantly found in the Brazilian states of Amazonas, Acre and Rondônia [20], in non-flooded areas [21]. This species is highly promising for açaí fruit extraction, as the state of Amazonas is already the second largest producer of Brazil [15]. Therefore, the establishment of strategies for *E*. *precatoria* domestication, conservation and management of populations is extremely necessary, in addition to the support of riverside communities and farmers to conserve this species based on their genetic diversity [15].

The fast growth of açaí (*E*. *oleracea* and *E*. *precatoria*) pulp production in the last decades and the threats of extinction for *E*. *edulis* indicate an urgent demand for new genomic resources in this genus, contributing to the understanding of the evolution and population dynamics of the species, which can result in the best planning of sustainable development and conservation [9, 15]. Fortunately, high-throughput sequencing technologies have revolutionized how genomic data can be obtained for plant species and have allowed the complete assembly of the chloroplast genome for an increasing number of non-model species [22–24], while providing useful information for the study of genetic variation of native species.

The common structure of the chloroplast genomes typically has four parts: two single copy regions, one large (LSC) and one small (SSC), and a pair of inverted regions (IRs) [25] which basically consist of long circular or linear molecules (120 to 180 kb), usually conserved across palms species [26]. Apart from the conservative nature and low nucleotide substitution rates of the chloroplast sequences [27], the description of this organellar genome is important to identify repetitive sequences and polymorphic regions, useful to further obtain molecular markers, which can be applied to assess genetic structure and diversity of natural populations [28, 29]. Whole chloroplast genomes and chloroplast sequences have been used to infer phylogenetic relationships and investigate diversification patterns [24, 30–32]. The tribe Euterpeae was already analyzed with combined chloroplast and nuclear sequence data [33], and whole chloroplast genomes have been reported in reference for two species in the *Euterpe* genus (*E*. *oleracea* and *E*. *edulis*) [34]. These priors research emphasize the differentiation of *E*. *edulis*, due to its distribution in a different Brazilian biome (Atlantic Forest biome). But, the addition of new chloroplast genomes can give more consistency to the evolutionary processes of the genus.

Moreover, the amount of plastid sequences present in a data from total genomic DNA extraction depends on different factors and can vary substantially, from 0.4% to 29.5% [23], and the recovery of these sequences can occur through the use of genome skimming. Therefore, the remaining nuclear and mitochondrial sequences can provide genomic resources [27, 35], representing useful information to study the genetic variation of native species.

Considering the effectiveness of genome skimming to produce genomic data, we employed this approach to obtain three newly sequenced complete chloroplast genomes, thereby facilitating the study of genetic diversity from *Euterpe* species. We were able to investigate: i) the plastid organization in *Euterpe* and the synteny level with other available Arecaceae species; ii) the repetitive sequences and polymorphic regions in the chloroplast genomes; iii) the Arecaceae family evolution based on a phylogenomic study with complete chloroplast genomes; and iv) the species divergence using the remaining nuclear and mitochondrial data from genome-skimming.

## Material and methods

### Leaf material

The leaf material from *Euterpe edulis* and *E*. *oleracea* were collected from the *ex situ* collection at the Escola Superior de Agricultura “Luiz de Queiroz” (ESALQ), Universidade de São Paulo (USP) in Piracicaba, São Paulo, Brazil (http://www.esalq.usp.br/trilhas/palm/) and the sample of *E*. *precatoria* was collected at the Instituto Nacional de Pesquisas da Amazônia (INPA), in Manaus, AM, Brazil. The collections were registered according to the Brazilian laws (SISGEN number A411583, Brazil).

### Intact chloroplast isolation in sucrose gradient and chloroplast DNA extraction

The chloroplast organelles were isolated using the sucrose gradient method [36]. About 20 g of fresh leaves from each species were frozen with liquid nitrogen and macerated. The material was resuspended in 200 mL of isolation buffer (50 mM Tris-HCl pH 8.0, 0.35 M sucrose, 7 mM EDTA, 5 mM 2-mercaptoethanol and 0.1% BSA) and incubated for 10 min in the dark. The suspension was filtered through two layers of Miracloth (Merck), and the filtrate was centrifuged at 1,000 × g for 10 min.

The pellet was resuspended in 5 mL of isolation buffer and the suspension slowly laid out in the density gradients of 20/45% sucrose in 50 mM Tris-HCl (pH 8.0), 0.3 M sorbitol and 7 mM EDTA. The gradients were centrifuged at 2000 × g for 30 min, and the green band formed at the interface containing intact chloroplasts were collected. The solution containing the chloroplasts were diluted in three volumes of buffer and centrifuged at 3,000 × g for 10 minutes to obtain the purified chloroplasts in the pellet.

The pellet was resuspended in 2% CTAB buffer to promote lysis. The suspension was incubated and stirred at 65°C for 1 h. The supernatant was extracted twice with an equal volume of chloroform: isoamyl alcohol (24: 1) and centrifuged at 10,000 × g for 20 min. An equal volume of isopropanol was added and incubated at 20°C for 1h. Finally, the aqueous phase was centrifuged for 10,000 × g for 20 min, and the chloroplast DNA (cpDNA) pellet was washed with ethanol (70%), dried and resuspended with 40μL TE (1 M Tris-HCl, 0.5 M EDTA, pH 8).

### Chloroplast genome sequencing, assembly and annotation

The genomic libraries were constructed using 100 ng of cpDNA and the Nextera DNA Flex kit (Illumina), following the manufacturer’s instructions. Paired-end sequencing (2x 150 bp) was performed on the Illumina NextSeq550 platform (Fundação Hemocentro de Ribeirão Preto, Brazil).

The assembly was conducted in three steps: First, the filtered reads from *Euterpe oleracea* were assembled in NOVOPlasty v 4.2 [37] (https://github.com/ndierckx/NOVOPlasty) using the *rbcL* gene sequence as a seed (NCBI accession number: MN621452.1) and the chloroplast genome of *Acrocomia aculeata* (NCBI accession number: NC_037084.1), a native Brazilian palm, as a reference to ordinate the contigs. Subsequently, all the three chloroplast genomes (*E*. *precatoria*, *E*. *edulis* and *E*. *oleracea*) were assembled in GetOrganelle v 1.7.3.1 [38] (https://github.com/Kinggerm/GetOrganelle/) using the *E*. *oleracea* chloroplast sequence obtained in NOVOPlasty as seed.

Briefly, in GetOrganelle, the chloroplast genomes were assembled using default settings, starting with the recruitment of target-associated reads using Bowtie2 [39]. In this step, the seed chloroplast genome was used as target for the extension in five iterations. Then, the total target-associated reads were *de novo* assembled into a fasta assembly graph using SPAdes [40]. The target-associated reads and the contigs in the assembly graph, with a contig label table created with BLAST hits, were used to exclude and minimizes non-chloroplast genome. To finalize, the Gaussian mixture distribution was used to determine coverage values of all contigs in the simplified assembly graph. In most cases of empirical plant genome skimming data, the chloroplast has significantly higher coverage than any other genome. Therefore, the coverage values of plastid, mitochondrial and nuclear contigs are expected to be classified into different Gaussian components. GetOrganelle deleted the contigs with coverage value far from the target coverage distribution with the EM (Expectation-Maximization) algorithm, semi-supervised learning and the weighted Gaussian mixture model [38].The *de novo* assemblies were trimmed and used to calculate all possible paths of a complete organelle genome [38]. Finally, the correctness and coverage of the assembly was assessed and confirmed in Geneious v2020 2.4. (https://www.geneious.com/, last assessed January, 2021). We used the “Map to reference” function to map the paired-end raw data onto the final assembled chloroplast genomes.

The chloroplast genome annotation was performed in GeSeq (Organellar Genome Annotation) [41] from the Chlorobox platform, with settings for the identification of protein coding sequence (CDS), rRNAs and tRNAs based on reference chloroplast sequences and homologies through BLAST search. Following the automatic annotation, a manual correction of start and stop codons and a verification of pseudogenes and intron positions were performed using GenomeView [42]. We then obtained the chloroplast circular genome maps using OGDRAW [43].

The chloroplast genome sequences were submitted to the NCBI GenBank database and can be accessed with the following accession numbers: *Euterpe edulis* (ON533738), *Euterpe oleracea* (ON533739) and *Euterpe precatoria* (ON533740).

### Chloroplast genome structure comparison

To perform a comparative study and access the synteny between the obtained chloroplast sequences, we used a Perl script of MUMmer4 [44] (https://github.com/mummer4/mummer) to align the chloroplast genome from *E*. *edulis*, *E*. *oleracea* and *E*. *precatoria* with the function NUCmer. This analysis enables the identification of the conserved regions among sequences between species. The results were visualized in dot plots created by the function MUMmerplot. Additionally, to compare and align the *Euterpe* chloroplast sequences obtained with the GenBank sequences [34], MAFFT v.7 [45] was used as a way to identify differences between them. The location of the additional sequences found between them was also annotated in GeSeq [41].

Taking into account the species relationships from Arecoideae subfamily, and the previous evidence from palms chloroplast genome structures [28, 46], we decided to conduct two multiple progressive sequence alignment in Mauve v.2.4.0 [47]. The first one only included chloroplast genomes from Brazilian native species of subfamily Arecoideae: *Euterpe edulis*, *Euterpe oleracea*, *Syagrus coronata*, *Astrocaryum aculeatum*, *Astrocaryum murumuru* and *Acrocomia aculeata*. The second analysis was carried out using 17 chloroplast genomes from different palm species (S1 Table) available in GenBank. Taking into the account the evolution of the group, we selected the species that represented the five palm subfamilies: *Phytelepas aequatoriallis* and *Pseudophoenix vinifera* (Subfamily: Ceroxyloideae); *Trachycarpus fortune* and *Caryota mitis* (Subfamily: Coryphoideae); *Nypa fruticans* (Subfamily: Nypoideae); *Calamus caryotoides* and *Eresmopatha macrocarpa* (Subfamily: Calamoideae); *Veitchia arecina* (Subfamily: Arecoideae) and the Brazilian native species also from the Arecoideae subfamily.

Expansions and contractions in the inverted repeats (IR) regions from the chloroplast genome structure were also explored. The chloroplast genome junctions (IRB/LSC; IRB/SSC, SSC/IRA; IRA/LSC) from the *Euterpe* species, and other Brazilian palm species from subfamily Arecoideae were examined to identify differences between individuals within the same genus and among subfamilies.

### Identification of SSRs, dispersed repeats, indels, SNPs and nucleotide divergence hotspots

Simple sequence repeats (SSR) consisting of 1–6 nucleotide units were carefully determined using the web package MISA (available at https://webblast.ipk-gatersleben.de/misa/) [48]. The criteria to search SSR motifs were: SSR of one to six nucleotides long, with a minimum repeat number of 10, 5, and 4 units for mono-, di-, and trinucleotide SSRs, respectively, and three units for tetra-, penta- and hexanucleotide SSRs. The SSRs sequences and location were compared among the species. The dispersed repeats (forward, reverse, palindrome and complement sequences) identified in REPuter [49] were based on the following criteria: minimum repetition size ≥ 30 bp and sequence identity ≥ 90% (Hamming distance = 3). Posteriorly, the position of the SSRs and repeats were manually compared with the gene annotation of each chloroplast genome.

MAFFT v.7 [45] was used to obtain pairwise alignments between the chloroplast genomes to pinpoint small insertions/deletions (indels) in the sequences. The alignment between the three species was also used to identify single nucleotide polymorphisms (SNPs) and nucleotide divergence hotspots with DnaSP v.5 [50]. Specific coding genes with a high number of SNPs were aligned with the software Muscle [51] in the Selecton Server [52], which was also used to identify the synonymous (Ka) or non-synonymous (Ks) mutations. With this analysis, according with the Ka/Ks ratios, it was possible to determine positive (Ka/Ks > 1) or purifying (Ka/Ks < 1) selection, estimated under the evolutionary model M8.

A sliding window analysis (window length of 200 bp and step size of 50 bp) was conducted to find nucleotide divergence hotspots. All the positions of indels, SNPs and divergence hotspots were manually identified using the annotation of the aligned chloroplast genomes, performed previously in GeSeq (Organellar Genome Annotation) [41].

### Phylogenomic studies

Plastid sequences of 54 palm species plus one outgroup (*Daypogon bromeliifolius*, Dasypogonaceae) (S1 Table) curated annotated features in Genbank format were separated into genes and entered into a SQLITE database using a custom Python script (available on demand from the corresponding author). Sequences of all putative coding proteins were then grouped by species and aligned with MAFFT, and later assembled into an interleaved NEXUS file for phylogenetic analyses. Species with missing sequences were filled with missing data in each gene matrix. Each block of gene sequences was then manually checked for start and stop codons and evidence of non-coding behaviour. For the coding genes, we annotated first, second and third positions for each codon, but the regions *cemA* and *rpl16* presented strange start codons and non-triplet insertions and were not annotated for codon positions. We tested three *ad hoc* partitioning schemes for models of molecular evolution: i) single model for the whole matrix, ii) four partitions: 1^st^, 2^nd^, 3^rd^ codon positions and a partition for *cemA* +*rpl16* (not split into codon positions), iii) five partitions: 1^st^, 2^nd^, 3^rd^ codon positions, *cemA* (not split into codon positions), *rpl16* (not split into codon positions). The assessment of molecular evolution model for each partition was calculated in the different partition schemes with the Akaike Information Criterion (AIC) in MrModelTest 2.0 [53], but they were all GTR+I+G, probably due to the complexity of mixing different genes in each partition. The assessment of the best partition scheme was then made using stepping stone (SS) [54] which is more accurate than taking the harmonic means of the likelihoods for model comparison. Bayesian inference were carried out with MrBAYES 3.2.7 [55] in the CIPRES platform [56], with one run of two chains, and 10 × 10^6^ generations, sampling one tree every 1,000. Marginal likelihoods of the SS runs were then compared using the standard Bayes Factors scale [57]. The same partition schemes were run separately to estimate the phylogeny with two runs of four chains (three hot and one cold), and 20 × 10^6^ generations, sampling one tree every 1,000 and discarding 25% initial generations for burn-in. The remaining trees were checked for estimated sample sized (ESS) > 200 in all parameters, and the final majority-rule was computed with MrBayes, rooted with the single outgroup *D*. *bromeliifolius* and the tree was exported to FigTree 1.4 [58] for drawing, and later improved with InkScape [59] for designing the figures.

### Genetic variation and comparative analyses with nuclear and mitochondrial sequences

After the assembly of the chloroplast genomes, the reads were also used to obtain SNPs for preliminary comparative analysis between species. Initially, the raw reads of each species were aligned in their respective assembled chloroplast genome using Bowtie2 [39]. Subsequently, the non-aligned sequences (non-chloroplast) were obtained with SAMTools [60], and converted to fastq file using Picard Tools program [61].

The reads corresponding to non-chloroplast genome (nuclear and mitochondrial data) were cleaned using *process_shortreads* from the Stacks package v.1.42 [62]. Sequences from each species were used to build loci with a minimum depth of three and up to two mismatches, applying the *ustacks* program (-m 3, -M 2) [62]. Subsequently, a catalog was built from each species, allowing for up to two mismatches (-n 2, cstacks) [62], and using *sstacks* [62] loci were matched with the catalog of each species. *Rxstacks* [62] was used as a correction step, assuming a mean log likelihood of -10 to discard loci with lower probabilities. The *populations* [62] program was administered for a final filtering to retain loci with a maximum missing data of 5%, and also used to verify the number of private alleles (*Ap*), observed heterozygosity (*H*_*O*_) and expected heterozygosity (*H*_*E*_; S1 Fig).

Finally, the mean sequence depth and the mutation counts of the SNPs were determined in vcftools [63]. The number of alleles (*A*) in each species and the genetic differentiation (*F*_*ST*_) between them were estimated with *Adegenet* v 1.3–1 [64] and *Genpop* v 1.1.7 [65] for the platform R v 4.0.3 [66].

## Results

### Organization of the three *Euterpe* species chloroplast genomes

**Fig 1:**
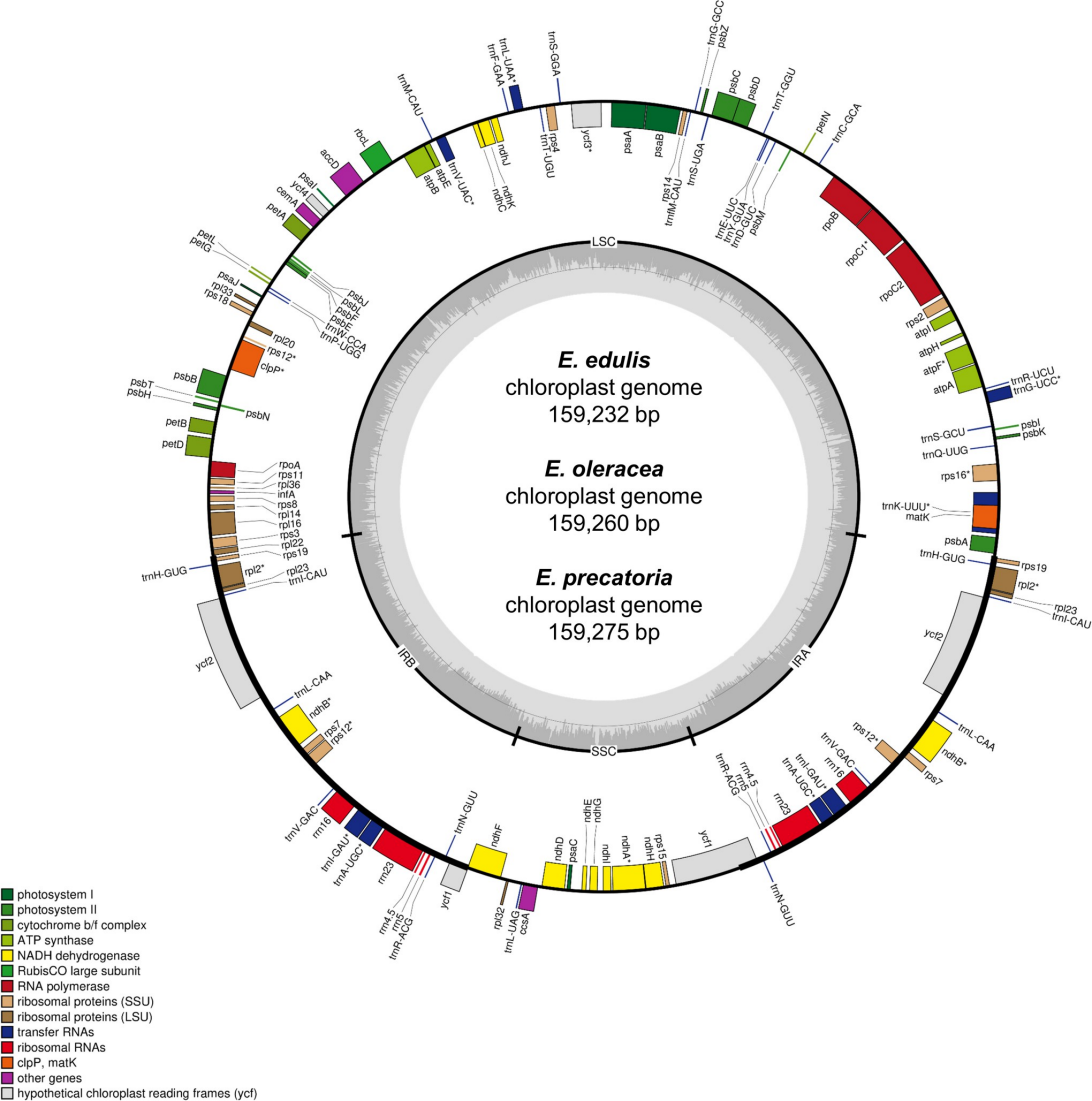
Gene map of the *Euterpe edulis*, *E*. *oleracea* and *E*. *precatoria* chloroplast genomes. Genes represented inside the large circle are oriented clockwise and the ones outside are oriented counter clockwise. The different colors represent functional groups, and the darker gray in the inner circle indicates the GC content. The quadripartite structure is also reported as: LSC = Large Single Copy, SSC = Small Single Copy, IRA/IRB = Inverted Repeats A and B.

The chloroplast genomes of *E*. *edulis*, *E*. *oleracea* and *E*. *precatoria* had the typical quadripartite structure (Fig 1), with the presence of two copies of inverted repeat regions (Inverted Repeats A and B = IRA and IRB) and two single copy regions (Large Single Copy = LSC and Small Single Copy = SSC). *E*. *precatoria* had the largest chloroplast genome size (159,275 bp) and the largest LSC and SSC (87,282 bp and 17,756 bp, respectively), while *E*. *edulis* had the largest IR regions and *E*. *oleracea* a slightly larger amount of GC content (37,3%) (Table 1). All three chloroplast genome annotations resulted in the identification of 113 unique genes, with 30 tRNAs, 4 rRNAs and 79 protein coding genes (Fig 1, Table 2).

**Table 1:** General features of chloroplast genomes of three *Euterpe* species.

Species	Total cpDNA size (bp)	Length of LSC region (bp)	Length of IR region (bp)	Length of SSC region (bp)	GC content (%)	Protein coding genes (bp)	tRNA coding genes (bp)	rRNA coding genes (bp)	Introns (bp)	Intergenic Regions (bp)
*Euterpe edulis*	159,232	87,237	54,280	17,715	37.20	80,462	2,880	9,052	18,068	48,770
*Euterpe oleracea*	159,260	87,273	54,232	17,755	37.30	80,415	2,881	9,052	18,062	48,850
*Euterpe precatoria*	159,275	87,282	54,234	17,759	37.20	80,411	2,881	9,052	18,064	48,867

**Table 2:** Gene content in *Euterpe edulis*, *E*. *oleracea* and *E*. *precatoria* chloroplast genomes according to each respective category.

Category	Gene							
Subunits of photosystem I	*psaA*, *psaB*, *psaC*, *psaI*, *psaJ*							
Subunits of photosystem II	*psbA*, *psbB*, *psbC*, *psbD*, *psbE*, *psbF*, *psbH*, *psbI*, *psbJ*, *psbK*, *psbL*, *psbM*, *psbN*, *psbT*, *psbZ*							
Subunits of cytochrome b/f complex	*petA*, *petB*^a^, *petD*^a^, *petG*, *petL*, *petN*							
Subunits of ATP synthase	*atpA*, *atpB*, *atpE*, *atpF*^a^, *atpH*, *atpI*							
Large subunit of rubisco	*rbcL*							
Subunits of NADH-dehydrogenase	*ndhA*^a^, *ndhB*^a^^,^^b^, *ndhC*, *ndhD*, *ndhE*, *ndhF*, *ndhG*, *ndhH*, *ndhI*, *ndhJ*, *ndhK*							
Proteins of large ribosomal subunit	*rpl2*^a^^,^^b^, *rpl14*, *rpl16*^a^, *rpl20*, *rpl22*, *rpl23*^b^, *rpl32*, *rpl33*, *rpl36*							
Proteins of small ribosomal subunit	*rps2*, *rps3*, *rps4*, *rps7*^b^, *rps8*, *rps11*, *rps12*^a^^,^^b^^,*c*^, *rps14*, *rps15*, *rps16*^*a*^, *rps18*, *rps19*^b^							
Subunits of RNA polymerase	*rpoA*, *rpoB*, *rpoC1*^a^, *rpoC2*							
Maturase	*matK*							
Translational initiation factor	*infA*							
Protease	*clpP* ^a^							
Envelope membrane protein	*cemA*							
Subunit of acetyl-CoA carboxylase	*accD*							
Cytochrome c biogenesis	*ccsA*							
Conserved hypothetical genes	*ycf3*^a^, *ycf4*							
Component of TIC complex	*ycf1*							
Component of 2-MD heteromeric AAAATPase complex	*ycf2* ^b^							
Ribosomal RNAs	*rrn4*.*5*^b^, *rrn5*^b^, *rrn16*^b^, *rrn23*^b^							
Transfer RNAs	*trnA–UGC* ^a^^,^^b^; *trnC–GCA*; *trnD*–*GUC*; *trnE–UUC*; *trnF–GAA*; *trnfM–CAU*; *trnG–UCC*^a^							
	*trnG–GCC*; *trnH–GUG*^b^; *trnI–CAU*^b^; *trnI–GAU*^a^^,^^b^; *trnK–UUU*^a^; *trnL–CAA*^b^; *trnL–UAA*^a^;							
	*trnL–UAG*; *trnM–CAU*; *trnN–GUU*^b^; *trnP–UGG*; *trnQ–UUG*; *trnR–ACG*^b^; *trnR–UCU*;							
	*trnS–GCU*; *trnS–UGA*; *trnS–GGA*; *trnT–UGU*; *trnT–GGU*; *trnV–GAC*^b^; *trnV–UAC*^a^;							
	*trnW–CCA*; *trnY–GUA*							

^a^Intron-containing gene

^b^Two gene copies in the Irs

^c^Gene divided into two independent transcription units.

Considering the duplicated genes in the IRs, more than 50% of the three chloroplast genome sequences are from protein coding regions (86 genes, Tables 1 and 2). Among genes, *ycf2* presented a slight difference between species, 6,903 bp in *Euterpe edulis* and 6,879 bp in *E*. *oleracea* and *E*. *precatoria*. Furthermore, chloroplast tRNA and rRNA were conserved among species, constituting 1.8% and 5.7% of their sequences, respectively. Introns constituted ca. 12% and intergenic regions represented almost 31% of the chloroplast genomes (Table 1).

All chloroplast genomes had 17 unique genes (11 protein- and six tRNA-coding genes) with introns, five duplicated genes in the inverted repeats regions with introns, and two introns in the *ycf3* and *clpP* genes. Accounting for the lengths, the largest intron was identified in *trnK–UUU* (2,621 bp–*E*. *edulis*; 2,615 bp–*E*. *oleracea*; 2,618 bp–*E*. *precatoria*) and the smallest in *trnL-UAA* (519 bp–*E*. *edulis*, 519 bp–*E*. *oleracea*, 521 bp–*E*. *precatoria*).

### Chloroplast genome structures and comparative analyses

The comparative analysis enabled us to identify a high level of synteny between the three *Euterpe* chloroplast genomes, with large conserved blocks. The only structural difference among *Euterpe* chloroplast genomes were three small inversions and single nucleotide polymorphisms (SNPs) between *E*. *edulis* and *E*. *oleracea* chloroplast sequences (S2A–S2C Fig). In a local alignment we detected these divergences in the region between 66,081 to 69,440 bp; the first corresponding to an intergenic region between *petA* and *psbJ* genes from *E*. *oleracea*; the second found in the *psbJ* gene and the third in the intergenic region of *trnW-CCA* and *trnP-UGG* from *E*. *oleracea* (S2D Fig).

The parallel between the chloroplast genomes of *Euterpe edulis* and *Euterpe oleracea* with the ones available in Genbank [34] showed differences in the total number of base pairs. *E*. *edulis* and *E*. *oleracea* were 835 bp and 458 bp, respectively, larger than the others. The alignment revealed that these increases were due to insertions that occurred mainly in intergenic spacers (83.3% of insertions in *E*. *edulis* and 100% in *E*. *oleracea*; S2 Table).

**Fig 2:**
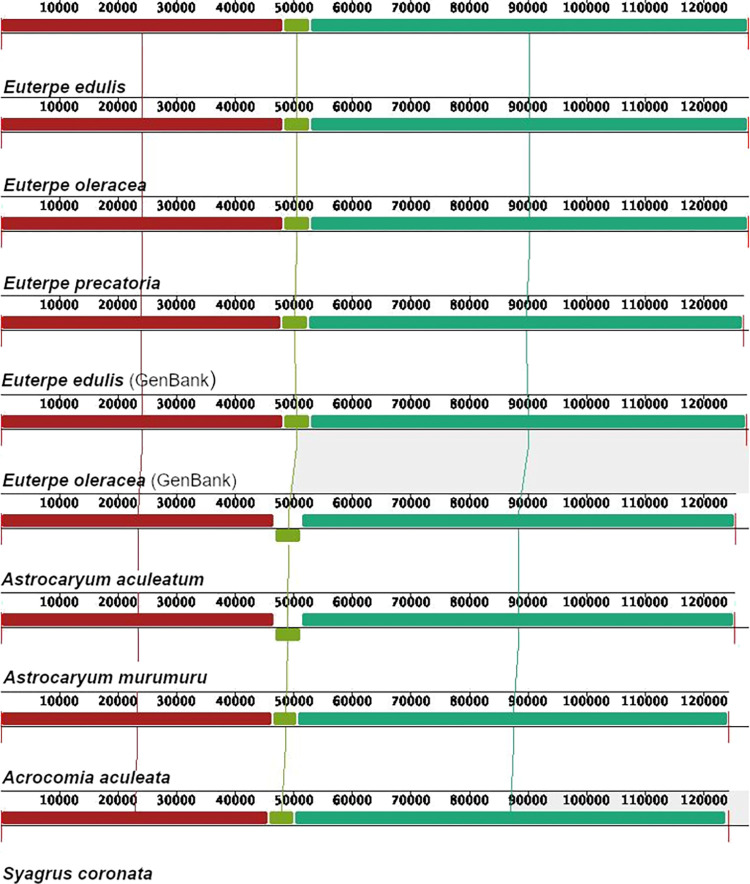
Synteny and divergence in the SSC size detected in Arecaceae chloroplast genomes using the Mauve multiple-genome alignment program. A sample of nine different chloroplast genomes is shown. Color bars indicate syntenic blocks and the lines indicate the correspondence between them. Blocks on the top row are in the same orientation, while blocks on the bottom row are in inverse orientation.

Although exhibiting high synteny and only a few structural rearrangements in their chloroplast genome, the most notable divergence among the three Brazilian native species from subfamily Arecoideae was in the length of the LSC, between 40,000 and 50,000 bp (Fig 2). A high level of synteny was also observed in a comparison of palm chloroplast genomes from 12 genera, with five subfamilies (S3 Fig). *Astrocaryum aculeatum*, *A*. *murumuru* and *Syagrus coronata* are native palms from subfamily Arecoideae with smaller LSCs compared with *Euterpe* palms (Fig 2 and S3 Fig). *Acrocomia aculeata*, also a Brazilian palm, presented a reduction in the size of the region comprising the genes *ndhJ*, *ndhK*, *ndhC*, *trnF-GAA* and *trnL-UAA* (in lime green Fig 2). Additionally, *Astrocaryum aculeatum* and *A*. *murumuru* showed a flip-flop recombination in this same region (in lime green, Fig 2) [67].

**Fig 3:**
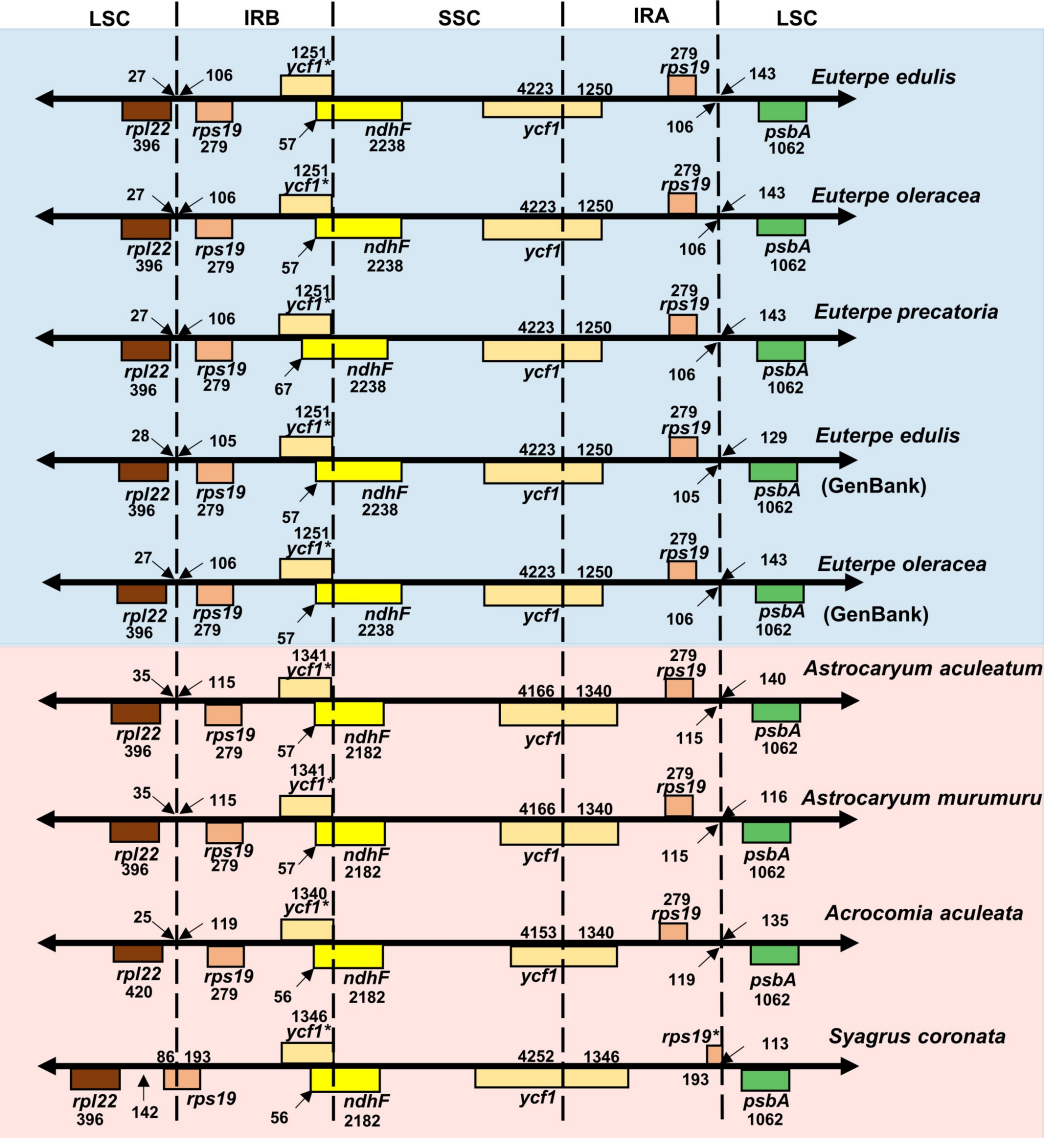
Comparison of the IRA and IRB borders among Brazilian palms from the Arecoideae species. The numbers indicate the lengths of IGSs, genes, and spacers between IR-LSC and IR-SSC junctions. The ycf1* and rps19* genes have incomplete CDSs.

In addition, we explored in more details the chloroplast IR expansions and contractions of the Brazilian palm species from the subfamily Arecoideae (Fig 3). Comparing the IR borders from Genbank available for *Euterpe edulis* chloroplast genome, it was possible to observe a small difference in the *rpl22*-*rps19* location and an increase in the length of *rps19*-*psbA* intergenic spacers. Among the chloroplast genomes from *Euterpe* genus, we identified an expansion in the *ndhF* gene at the IRB region from *Euterpe precatoria*. Among the Brazilian palm species, a contraction in the IR from *Syagrus coronata* influenced the position of *rps19*. Among all species analyzed, the most common variations were identified around the positions of *rpl22-rps19* (IRB-SSC), the *ycf1* length (IRB and IRA), and the *rps19-ycf1* (IRA-LSC) (Fig 3).

### Sequence repeats and polymorphisms between *Euterpe* chloroplast genomes

**Fig 4:**
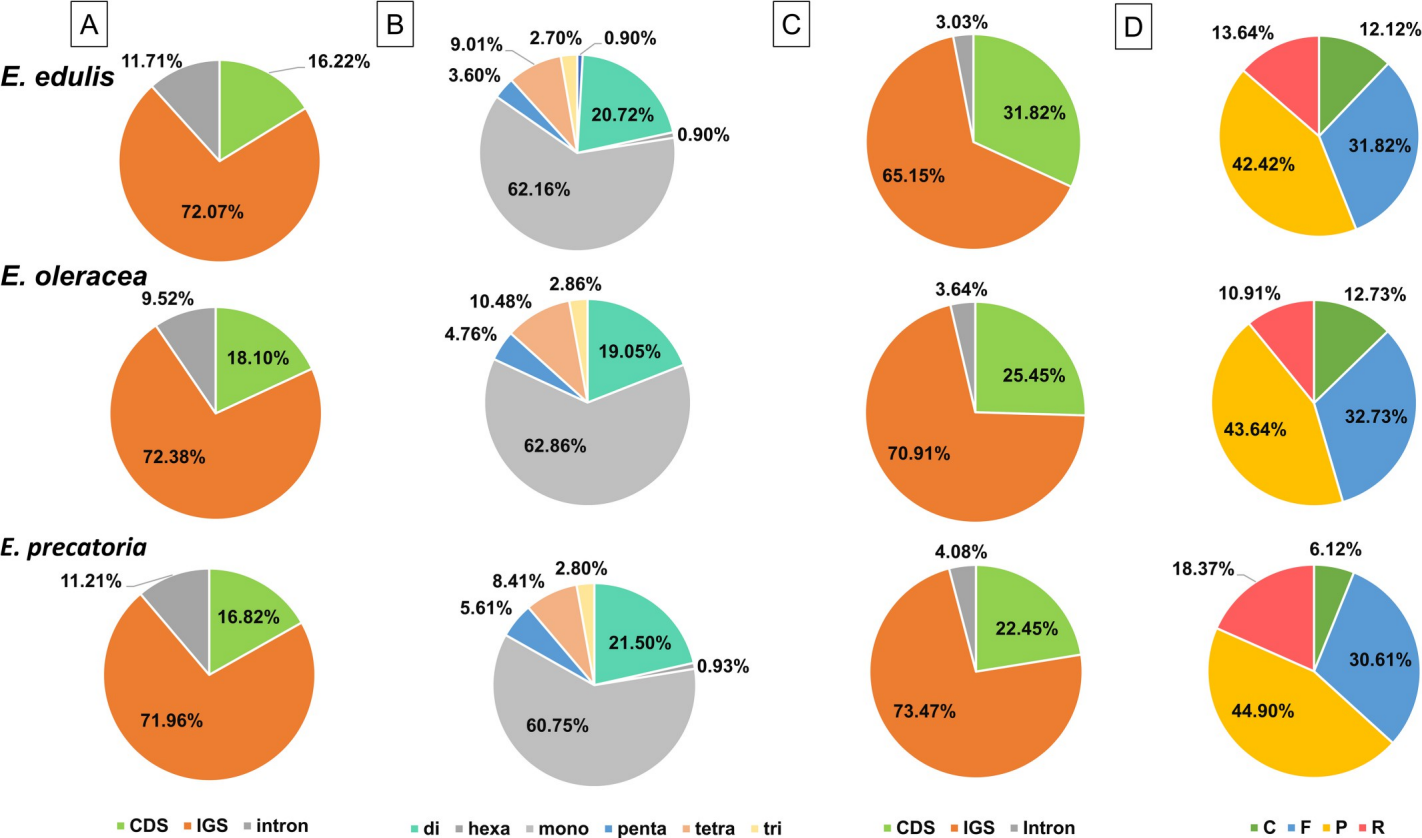
Distribution and classification of SSR and dispersed repeats in the chloroplast genomes of *Euterpe edulis*, *E*. *oleracea* and *E*. *precatoria*. (A) Proportion of coding and non-coding regions containing SSRs; (B) Proportion of different types of SSR present in the chloroplast genomes; (C) Proportion of regions containing repeats; (D) Frequency distribution of different types of repeats: F = Forward, P = Palindrome, R = Reverse and C = Complement. CDS = Coding sequence, IGS = Intergenic spacer.

We identified a total of 323 SSRs (*E*. *edulis*: 111, *E*. *oleracea*: 105, *E*. *precatoria*: 107 SSRs) in the chloroplast genomes from the three species. Most of them were located in intergenic spacer regions (IGS: 72.07% *E*. *edulis*, 72.38% *E*. *oleracea*, 71.96% *E*. *precatoria*; Fig 4A), especially in the IGS between the tRNAs trnS-GCU/trnG-UCC (S3 Table). The SSRs were more abundant in the LSC region of the chloroplast genomes (78.38% *E*. *edulis*, 78.10% *E*. *oleracea*, 77.57% *E*. *precatoria*; S3 Table) and less frequent in the IR regions. They were mostly mononucleotides (62.16% *E*. *edulis*, 62.86% *E*. *oleracea*, 60.75% *E*. *precatoria*; Fig 4B) and composed with A/T motifs (60.36% *E*. *edulis*, 60.00% *E*. *oleracea*, 59.81% *E*. *precatoria*; S4A Fig). From the 323 SSRs, 47 had the same sequence and gene location among *E*. *edulis*, *E*. *oleracea* and *E*. *precatoria*. We also identified 62 SSRs between *E*. *edulis/E*. *oleracea* and *E*. *edulis/E*. *precatoria*, and 68 between *E*. *oleracea and E*. *precatoria* (S4 Table).

Considering the dispersed repeats, we observed 66 in *E*. *edulis*, 55 in *E*. *oleracea* and 49 in *E*. *precatoria*, ranging from 30 to 77 bp (S4B Fig). These repeats were distributed mostly in the IGS regions (65.15% *E*. *edulis*, 70.91% *E*. *oleracea*, 73.47% *E*. *precatoria*; Fig 4C). Comparing the species, in *E*. *precatoria*, most of the repeats were found in the intergenic region of *psaC*/*ndhE*. However, in *E*. *edulis* and *E*. *oleracea* the repeats were concentrated in the *ycf2* gene (S5 Table). Regarding repeat types, most of the repeats are palindrome or forward sequences, 40% and 30%, respectively (Fig 4D). They were also mainly found in the LSC region (48.48% *E*. *edulis*, 52.73% *E*. *oleracea*, 44.90% *E*. *precatoria*; S5 Table).

**Fig 5:**
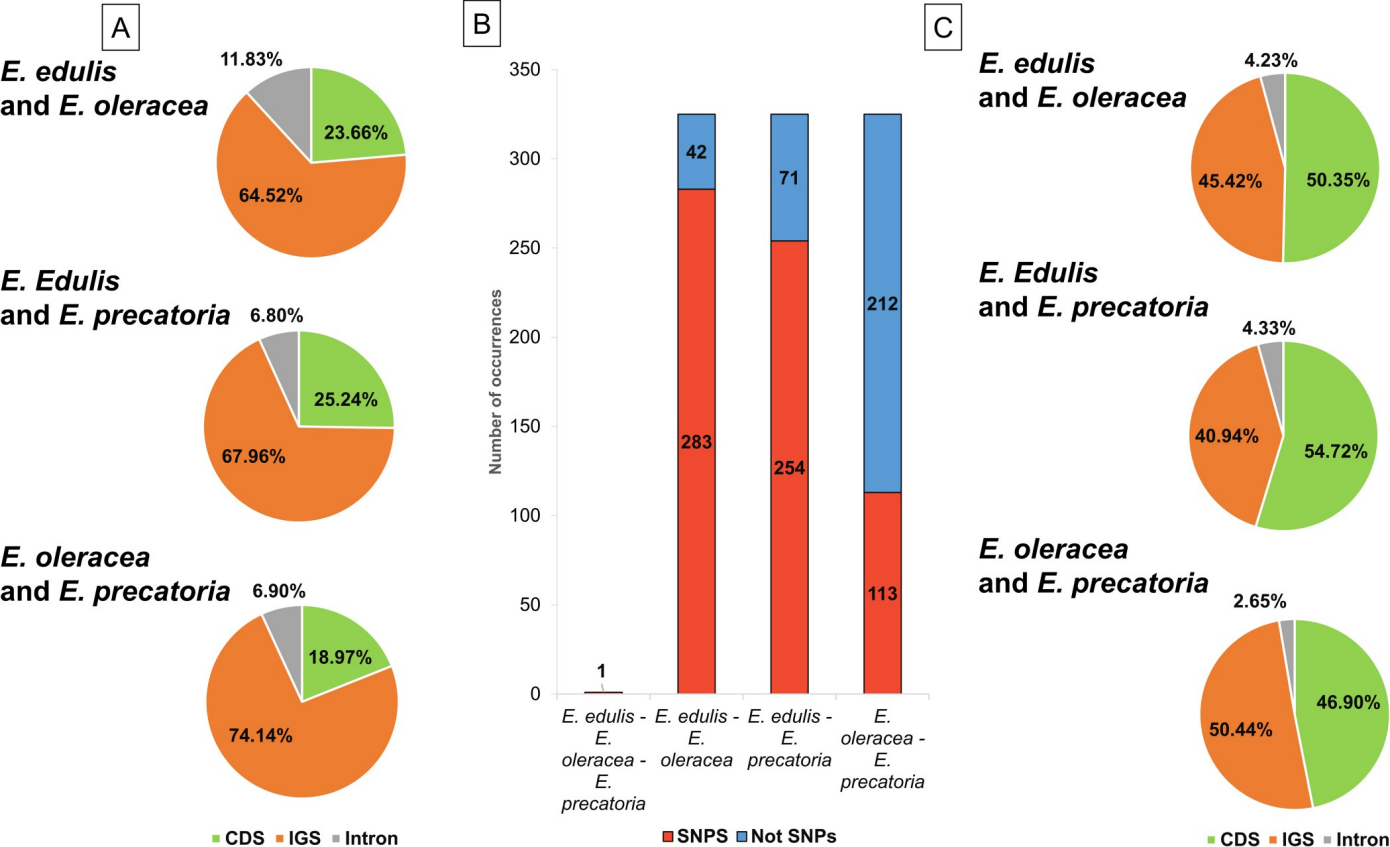
Indels and single nucleotide polymorphisms (SNPs) detected in comparisons between *Euterpe* chloroplast genomes. (A) Proportion of indels in different coding and non-coding regions; (B) Comparison of the number of SNPs found in the alignment (C) Proportion of SNPs in different coding and non-coding regions of the chloroplast genomes.

Based on the pairwise alignment between the species chloroplast genomes, we identified 93 indels between *E*. *edulis*/*E*. *oleracea*, 103 between *E*. *edulis/E*. *precatoria* and 58 between *E*. *oleracea*/*E*. *precatoria*. Most indels were distributed in the IGS (Fig 5A), especially between the *E*. *oleracea/E*. *precatoria* chloroplast genome, located mainly in the LSC region (80.65% ~ 72.41%; S6 Table). The highest occurrence of indels was identified in the *trnS-GCU*/*trnG-UCC* of the three alignments (S6 Table).

A total of 325 SNPs were detected between the three species (Fig 5B). In a pairwise comparison, 283 SNPs were detected between *E*. *edulis/E*. *oleracea* and 254 SNPs between *E*. *edulis/E*. *precatoria*, which were most frequent in the CDS regions (50.35% and 54.72%, respectively; Fig 5C). Between *E*. *oleracea/E*. *precatoria*, a lower number of SNPs was found (113 SNPs), having the greatest number concentrated in the IGS (50.44%; Fig 5C). Only one SNP was found shared among the three species (Fig 5B).

The large number of SNPs in the CDS region occurs mainly in the *atpE*, *ycf1* and *psbJ* genes (S7 Table). These *atpE*, *psbJ* and *ycf1* genes presented respectively 19, 18 and 18 SNPs for *E*. *edulis/E*. *oleracea* and 20, 19 and 21 SNPs for *E*. *edulis/E*. *precatoria*. For *E*. *oleracea/E*. *precatoria* there were only 1 (*atpE*) and 7 (*ycf1*) SNPs (S7 Table).

The LSC region had the highest number of SNPs in the chloroplast genomes (69.01% *E*. *edulis/E*. *oleracea*, 66.54% *E*. *edulis/E*. *oleracea*, 64.91% *E*. *oleracea/E*. *precatoria*; S7 Table). For *E*. *edulis/E*. *oleracea* the greatest number of SNPs corresponded to C/T—T/C (74) substitutions, the same was saw for *E*. *edulis/E*. *precatoria* T/C—C/T (66), and 29 substitutions of G/A—A/G and C/T—T/C was observed for *E*. *oleracea/E*. *precatoria* (S5 Fig).

**Fig 6:**
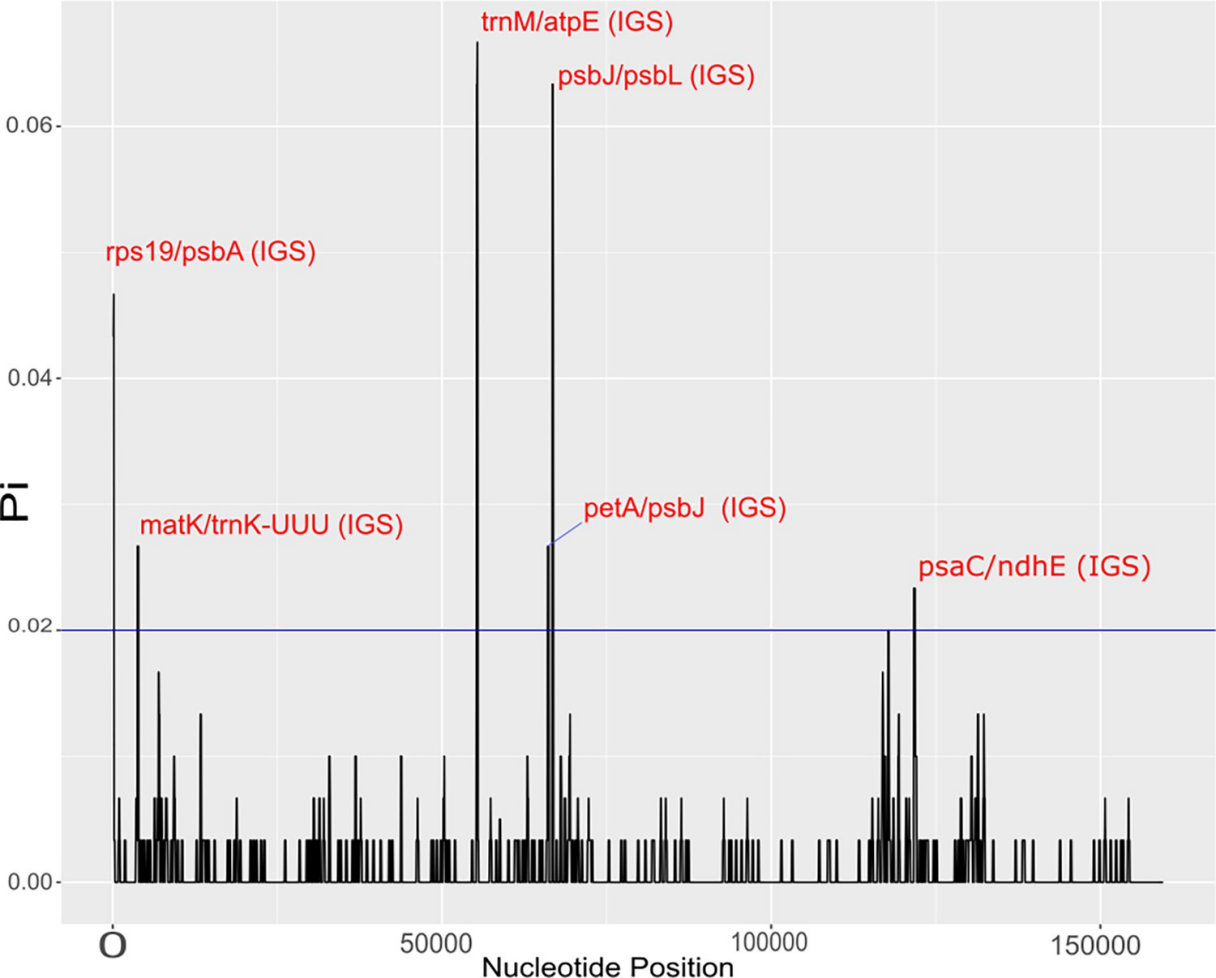
Sliding window analysis of the alignment from the chloroplast genome of *Euterpe edulis*, *E*. *oleracea* and *E*. *precatoria* chloroplast genomes. The regions with high nucleotide variability (Pi > 0.02) are indicated. Pi is the nucleotide diversity of each window, and the window length was 200 bp with 50 bp step sizes.

Considering pi > 0.02, the sliding window analysis revealed six hotspots of high nucleotide polymorphism among the *E*. *edulis*, *E*. *oleracea* and *E*. *precatoria* chloroplast genome sequence. The six hotspots were in IGS regions (Fig 6). Among them, the two hotspots with the highest polymorphism were located between the *trnM*/*atpE* (Pi > 0.06; Fig 6) and *psbJ*/*psbL* (Pi > 0.06; Fig 6). Comparing with the SNP identification, the *atpE* gene had the highest number of SNPs among the species *E*. *edulis/E*. *oleracea* and *E*. *edulis/E*. *precatoria*. The *psbJ* gene had 18 SNPs in *E*. *edulis/E*. *oleracea* and 19 SNPs in *E*. *edulis/E*. *precatoria*.

Both genes, *atpE* and *psbJ*, were analyzed for positive or purifying selection across the palm phylogeny using the estimation of Ka/Ks ratio. In both cases, the Ka/Ks < 1 revealed that no positively selected sites were found in the corresponding protein sequences, indicating purifying selection. The analysis of the amino acid sequence resulted in Ka/Ks rates varying from 0.17 to 0.87 for *atpE* (S8 Table) and ranging from 0.50 to 0.96 for *psbJ*. Also, from the 134 amino acids of the *atpE* protein, 68 had Ka/Ks < 0.19 and from the 40 amino acids, 37 had Ka/Ks < 0.55, in the case of *psbJ* (S9 Table).

### Phylogenomic of *Euterpe* based on chloroplast sequences

In the phylogenomic analysis, the marginal mean likelihoods of partition schemes for the two runs were i) -186887.39, ii) -178507.00, and iii) -178497.59. Therefore, the best partition schemes were by far the ones considering codon positions, although ‘iii’ was strongly better than ‘ii’ with the 2*log difference = 18,82 (>10 is strong in Kass and Raftery 1995). We also compared the majority-rule consensus trees of the three partition scheme analyses, which did not differ in topology, but in general the scheme ‘iii’ presented better support in some nodes. For that reason, the figure presented and used in our discussion will be based on partition scheme ‘iii’.

**Fig 7:**
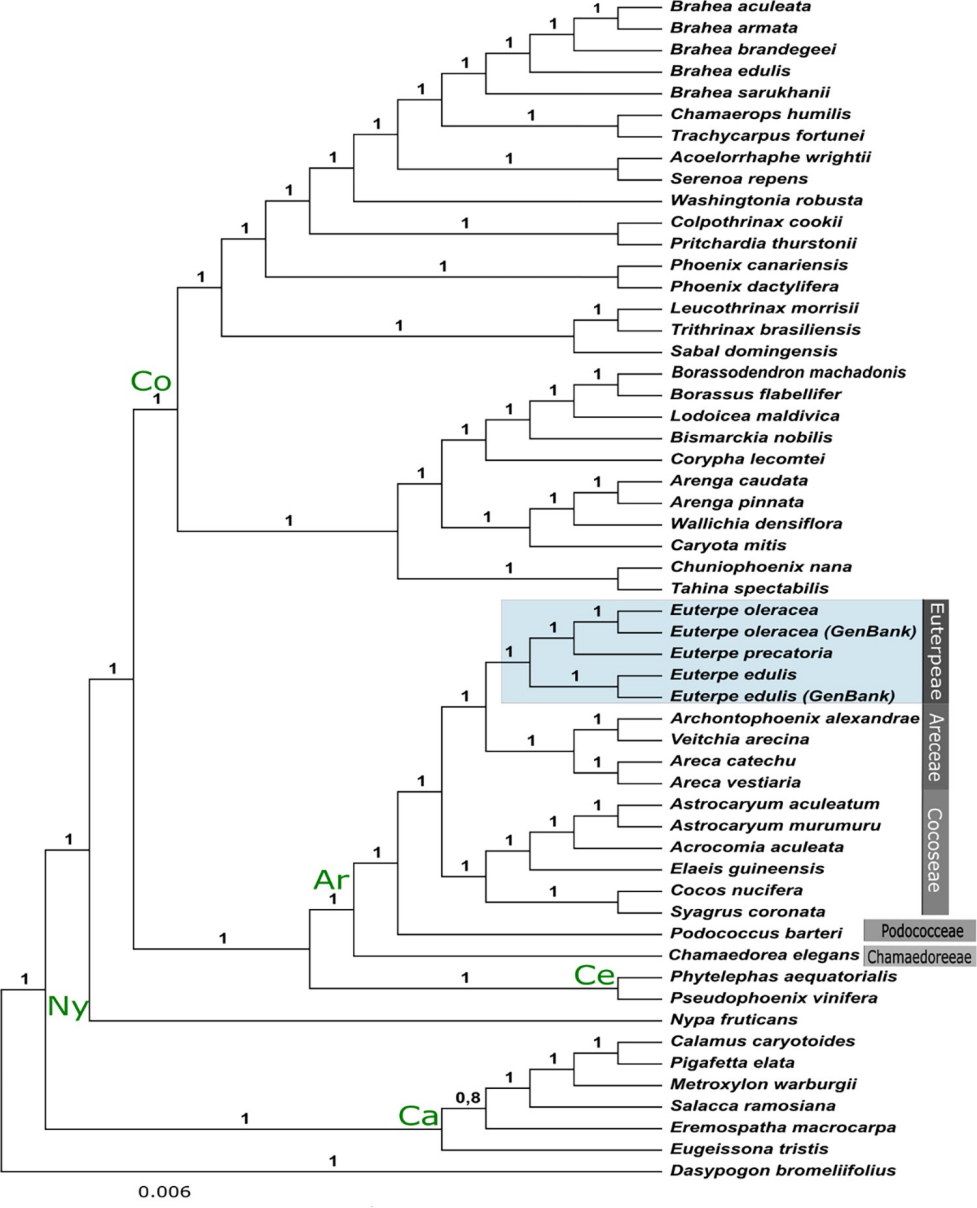
Majority-rule consensus tree of 30,000 trees obtained from a Bayesian inference analysis of chloroplast protein coding genes of 55 taxa. Posterior probabilities (PP) for each are indicated above branches. Co = Coryphoideae, Ar = Arecoideae, Ny = Nypoideae, Ce = Ceroxyloideae, Ca = Calamoideae.

The phylogeny using coding sequences from 54 whole chloroplast genomes from palm species rooted in the outgroup species *Dasypogon bromeliifolius* revealed the same relationships in subfamilies previously reported [34], with highly supported nodes (Fig 7). According to our results, the species from *Euterpe* were placed in subfamily Arecoideae, tribe Euterpeae, and were sister to tribe Areceae. The new samples of *E*. *oleracea* and *E*. *edulis* were grouped with the respective previously published chloroplast genomes and the *E*. *precatoria* was placed in a branch sister to *E*. *oleracea*.

### Species divergence with nuclear and mitochondrial genome

From the 11,738,792 reads that were generated for *E*. *edulis*, 553,857 (4.71%) aligned to the assembled chloroplast genome. The remaining (ca. 95%) reads correspond to nuclear and mitochondrial regions. Similar proportions were also found for *E*. *oleracea* and *E*. *precatoria* for which 12,987,827 and 12,612,001 reads were generated, respectively. Moreover, 249,387 (1.92%) and 132,227 (1.04%) were aligned to the assembled chloroplast sequence for both species, respectively. Using Stacks, we found 1,077 SNPs in the nuclear and mitochondrial genome sequences, with 10X mean sequencing depth per locus (S6A Fig). Among *Euterpe* species, more transitions than transversions were detected, and the most frequent mutations were A-G and C-T transitions (S6C Fig).

The highest number of alleles (*A* = 1,309) and highest observed heterozygosity (*H*_*O*_ = 0.162) was identified in *E*. *oleracea*, while the highest number of private alleles was observed in *E*. *precatoria* (*Ap* = 421). All the three species had a higher value of *H*_*O*_ compared with the expected heterozygosity (*H*_*E*_, Table 3).

**Table 3:** Parameters of genetic diversity using 1,077 SNPs found the nuclear and mitochondrial genome sequences from three *Euterpe* species.

	*E*. *edulis*	*E*. *oleracea*	*E*. *precatoria*
*A*	1,251	1,309	1,263
*Ap*	292	260	421
*H*_*O*_	0.162 (±0.011)	0.215 (± 0.013)	0.173 (±0.012)
*H*_*E*_	0.081 (±0.006)	0.108 (±0.006)	0.086 (±0.006)

*A* = number of alleles, *Ap* = number of private alleles, *H*_*O*_ = observed heterozygosity, *H*_*E*_ = expected heterozygosity.

The pairwise *F*_*ST*_ showed high and significant divergence between species, with values higher than 0.770. Although all the *F*_*ST*_ values were high, the species *E*. *edulis* and *E*. *precatoria* presented the greatest genetic divergence (0.860, S7 Fig) among the three pairs of species, and *E*. *edulis* and *E*. *oleracea* had the lowest divergence (0.779, S7 Fig).

## Discussion

### High level of conservation between Brazilian palms chloroplast genomes

Among the chloroplast genomes from the *Euterpe* genus, we found a conserved organization and gene content, with small differences between species, as in the length of the duplicated *ycf2* gene. The *ycf2* is the largest plastid gene reported in angiosperms [68], and is part of the 2-MD heteromeric AAA-ATPase complex directly interacting with various translocating preproteins [69]. Particularly, *ycf2* has an insertion in the *E*. *edulis* chloroplast genome that is absent in *E*. *oleracea* and *E*. *precatoria*, a feature previously observed in *Euterpe* chloroplast genomes [34]. Across palm species, the chloroplast genome structure is highly conserved as reported in other studies [28, 34, 70]. The multiple alignments with 15 different palms from five subfamilies did not exhibit significant rearrangements in the chloroplast structure. The only rearrangement observed was an inversion of 4.6 kb in the *Astrocaryum* chloroplast genome. It was possibly to have a lineage-specific structural variation, creating a isoform from a flip-flop recombination between inverted repeats [67]. However, differences in the total size of chloroplast genomes were observed in the same species, such as in *E*. *edulis* and *E*. *oleracea*, which may be due to the assembly method used. Different from the approach of all possible paths’ calculation to complete target organelle genome, as applied by GetOrganelle, other widely used pipelines utilize reference genomes to select and filter scaffolds/contigs for further concatenation or post-assembly gap filling and closing [38].

Besides these conserved general features, similar occurrences of SSRs were identified among the three *Euterpe* species. Currently, SSR markers are very useful in studies of population structure, genetic mapping, and evolutionary processes [71]. The SSR identified here can be a valuable resource for studies aiming at the conservation of the species or their sustainable exploitation. These SSR are mostly composed of A/T motifs, and are more frequently located in the IGS of the single-copy regions from the chloroplast genomes [1, 72, 73], which is expected since these regions evolve faster than the CDS [74]. Also, the limited occurrence of SSRs in the IRs is related to a lower mutation rate observed in these regions, caused by an efficient mechanism of gene copy-correction, as observed in other palm chloroplast genomes [34, 75].

The dispersed repeats (forward, palindrome, reverse and complement) showed the same pattern of the SSRs and were frequently distributed in the IGS of the single copy regions, differing from a predominant occurrence of reverse repeats (18,37%) in *E*. *precatoria*. Actually, the number of repeats found in the *Euterpe* chloroplast genomes were not considered high [76]. According to Milligan et al. [77], repetitive sequences are substrates for recombination and chloroplast genome rearrangements. Since non-structural rearrangements were detected in the *Euterpe* chloroplast genomes, low frequency of repetitive elements was also expected.

Regarding the structure patterns of the IRs, we could identify most of the variation related to expansion/contractions when comparing only Brazilian palm species from subfamily Arecoideae. Events of expansion and contraction at the IR boundaries was previously observed in the *ycf1* gene at IR-SSC/LSC junction and *rps19* intergenic spacer with *rpl22*/*psbA* in IR-SSC/LSC junctions of other species [28, 75].

### Indels and single nucleotide polymorphisms clearly demonstrate differentiation among the three *Euterpe* species, which was reflected in the phylogeny

We identified six hotspots of high nucleotide polymorphism with the largest polymorphism (Pi > 0.06) in intergenic regions composed mainly by genes with a great number of SNPs. The genes *atpE* and *psbJ* had the greatest number of SNPs, especially when considering the nucleotide substitutions between *E*. *edulis* and the *Euterpe* Amazonian species, *E*. *oleracea* and *E*. *precatoria*. This divergence between *E*. *edulis* in relation to the two Amazonian species was also observed in the number of indels and plastid SNPs and was almost 50% higher than the number of polymorphisms detected in a pairwise analysis with *E*. *oleracea* and *E*. *precatoria*.

Indels display no ambiguity in complex mutation patterns and SNPs are the most abundant type of markers [78], giving us a clear pattern of divergence between *E*. *edulis* in relation to *E*. *oleracea* and *E*. *precatoria*. In our study, these mutations were frequently found in the CDS than in the intergenic spacers. This high number of indels and SNPs between *E*. *edulis/E*. *oleracea* had already been identified in a previous study [34], but they were mainly found in non-coding regions, which presumably may be related to a more recent event of divergence.

Regarding the phylogenetic relationships among the palm species, we identified a clear distinction among subfamilies using the informative chloroplast coding sequences, corroborating previous studies [28, 34, 67, 79]. Our phylogeny suggests that *E*. *oleracea* and *E*. *precatoria* are sister species with an early split of *E*. *edulis* from these two, differently from what was observed previously, having four nuclear low copy markers and one plastid region [33]. Our result reveals the possibility of incongruences between nuclear and plastid phylogenies in *Euterpe*, since the previous study using nuclear markers reported paraphyly in *E*. *precatoria* and showed one of the *E*. *precatoria* varieties as sister to *E*. *edulis* [33].

The closest relationship between *E*. *oleracea* and *E*. *precatoria* could reflect their environmental occupation since both species occur in the Brazilian Amazon. *E*. *edulis*, nonetheless, evolved via vicariant split from its common ancestor (around 1.4 Mya), and the speciation was intensified by the savanna barrier composed from the Cerrado and Caatinga biomes [33, 34, 80]. Furthermore, the phenotypic plasticity and local adaptation may have favored the successful expansion of *E*. *edulis* throughout the Atlantic Forest [11, 80].

Regarding the coding sequences, the *rpl22*, *rpl32*, *rpl14*, *rps14*, *matK*, *accD*, *rbcl*, *ccsA*, *ycf2 and ycf1* genes have been reported to harbor positive selection in an analysis using 41 species of the Arecaceae family, including the *E*. *oleracea* and *E*. *edulis* chloroplast genomes [34]. This was highlighted as indicative of convergent evolution, associated with environmental adaptation in *E*. *edulis*. However, using 52 palm species, we could only identify a tendency of purifying selection for *psbJ* and *atpE*, the genes with higher SNPs and polymorphisms hotspots. This may reflect the typically conservative nature of the chloroplast genome sequences across most angiosperms [81, 82]. These genes are essential for plant development, since *atpE* encodes a subunit of the ATP synthase complex that participates in the photosynthetic phosphorylation [83] and *psbJ* is associated with photosystem II (PSII) [84].

### A comparison using nuclear and mitochondrial genome sequences detected high divergence between *Euterpe* species

The reads from nuclear and mitochondrial DNA obtained from the sequencing were used in this first-time comparison of the genetic diversity and genomic divergence among the three species from the *Euterpe* genus. The advances in next-generation sequencing (NGS) is allowing the discovery of a large number of single nucleotide polymorphism (SNPs), even for non-model species [85–88]. This generates a valuable source of bi-allelic genetic markers that are appropriate to evaluate the genetic diversity even when sample sizes are small [89]. Additionally, SNPs occurs at higher density across the genome, with lower genotyping error rates than other markers [90], enabling robust estimates of genetic diversity and structuring [9].

The three *Euterpe* species had similar number of alleles and low observed heterozygosity. Yet, they are higher than the expected heterozygosity, with *E*. *oleracea* presenting slightly higher results. However, despite this enlightening information obtained with the remaining nuclear and mitochondrial sequences, we highlight that these findings could be biased because they were based on only one individual per species. Considering assessments of genome-wide diversity, previous studies identifying SNPs in palms were only performed for *E*. *edulis* [9, 80]. Furthermore, the genetic diversity estimates from our *E*. *edulis* sample, that was also from the Atlantic rainforest of São Paulo state, were in agreement with previously published studies. This observed genetic variability could be influenced by the type of vegetation and the altitude where the samples were collected [9, 80].

Plastid genomes, unlike most nuclear chromosomes, are typically uniparentally inherited, with absence of recombination [74]. In our phylogeny, the species *E*. *edulis* is closer to *E*. *precatoria* than to *E*. *oleracea*. However, the *F*_*ST*_ results with nuclear and mitochondrial genomes show the greatest divergence between *E*. *edulis* and *E*. *precatoria*. Besides the fact that which analysis characterized the genetic and phylogenetic relationships among species, the results can be a consequence of the haploid nature of the chloroplastidial genome. This reduces genetic variability and can generate differences in the relationship between species when considering the nuclear genome. Also, for the phylogeny, only conserved protein-coding genes were used, whereas for the F_ST_ all type of non-chloroplast sequence were considered.

In summary, our results open the way for future genome-skimming studies in palms. Also, the addition of larger sample sizes from different sampling locations will provide a better understanding on the evolution and diversification of this important group of plants. However, the high differentiation found between the three *Euterpe* species may indicate that they are evolving independently and that this differentiation is caused by a private pool of alleles from each species.

## Supporting information

S1 Fig Flowchart of the SNPs identification methodology using nuclear and mitochondrial genomes sequences. (TIF)

S2 Fig Dot-plot analyses comparing three *Euterpe* chloroplast genomes. (A) *Euterpe edulis* (X–axis) x *Euterpe Oleracea* (Y-axis), (B) *E*. *edulis* (X-axis) x *Euterpe precatoria* (Y-axis), (C) *E*. *oleracea* (X–axis) x *E*. *precatoria* (Y-axis). The positive slope, in purple, represents the pair of sequences aligned and in the same orientation. The negative slope, in blue, represents the pair of sequence aligned, but in opposite orientation. The blue arrow in A highlights the region with inversions and SNPs between *E*. *edulis* and *E*. *oleracea*. (D) Local alignment with the chloroplast genomes of *E*. *edulis*, *E*. *oleracea* and *E*. *precatoria* in the region where inversions and SNPs were detected in A.
(TIF)

S3 Fig Synteny and divergence in the size of the SSC detected in Arecaceae chloroplast genome sequences. A sample of 20 different chloroplast genomes is shown. Color bars indicate syntenic blocks and the lines indicate the correspondence between them. Blocks on the top row are in the same orientation, while blocks on the bottom row are in inverse orientation.
(TIF)

S4 Fig SSR and dispersed repeats in the chloroplast genomes of *Euterpe edulis*, *E*. *oleracea* and *E*. *precatoria*. (A) The number of SSR motifs found in the three chloroplast genomes, considering sequence complementarities; (B) Number of dispersed repeats present in different size classes.
(TIF)

S5 Fig Number and type of SNPs found in the alignment of three *Euterpe* chloroplast genomes. (TIF)

S6 Fig Description of 1,077 SNPs found among the nuclear and mitochondrial genome sequences from three *Euterpe* species. (A) Mean depth per loci; (B) Mean depth per species sequenced; (C) Mutation count in the present in the 1,077 SNPS.
(TIF)

S7 Fig Pairwise *F*_*ST*_ based on 1,077 SNPs of nuclear and mitochondrial genomes sequences from three *Euterpe* species. (TIF)

S1 Table List of complete chloroplast genomes used in comparative analysis. Synteny and rearrangement, border comparison and phylogenomics.
(XLSX)

S2 Table Insertions identified in the alignment of *Euterpe edulis* and *E*. *oleracea* chloroplast genomes with sequences from GenBank (de Santana Lopes et al. 2021). CDS = Coding sequence; IGS = intergenic spacer; LSC = Large single copy; SSC = Small single copy.
(XLSX)

S3 Table Simple sequence repeats (SSR) identified in the complete chloroplast genomes of three *Euterpe* species. CDS = Coding sequence; IGS = intergenic spacer; LSC = Large single copy; SSC = Small single copy; IRs = Inverted Repeats.
(XLSX)

S4 Table Simple sequence repeats (SSR) among the chloroplast genomes of *Euterpe edulis*, *E*. *oleracea* and *E*. *precatoria* according to their location. (XLSX)

S5 Table Repeats identified in the chloroplast genomes of *Euterpe edulis*, *E*. *oleracea* and *E*. *precatoria*. F = Forward; P = Palindrome; R = Reverse; C = Complement; CDS = Coding sequence; IGS = intergenic spacer; LSC = Large single copy; SSC = Small single copy; IRs = Inverted Repeats.
(XLSX)

S6 Table Indels identified in the pairwise comparison of three *Euterpe* species complete chloroplast genomes. CDS = Coding sequence; IGS = intergenic spacer; LSC = Large single copy; SSC = Small single copy; IRs = Inverted Repeats.
(XLSX)

S7 Table Single nucleotide polymorphisms (SNPs) identified in the pairwise comparison of three *Euterpe* species complete chloroplast genomes. CDS = Coding sequence; IGS = intergenic spacer; LSC = Large single copy; SSC = Small single copy; IRs = Inverted Repeats.
(XLSX)

S8 Table Bayesian selection results based on synonymous (Ka)/non-synonymous (Ks) ratio of amino acid substitutions from the *atpE* gene in 54 palm species. (XLSX)

S9 Table Bayesian selection results based on synonymous (Ka)/non-synonymous (Ks) ratio of amino acid substitutions from the *psbJ* gene in 54 palm species. (XLSX)
