# Supplementary figures and images for: Complete chloroplast genomes and phylogeny in three Euterpe palms (E. edulis, E. oleracea and E. precatoria) from different Brazilian biomes
Source: PLoS One. 2022 Jul 28;17(7):e0266304. doi: 10.1371/journal.pone.0266304 (PMC9333295; doi:10.1371/journal.pone.0266304)

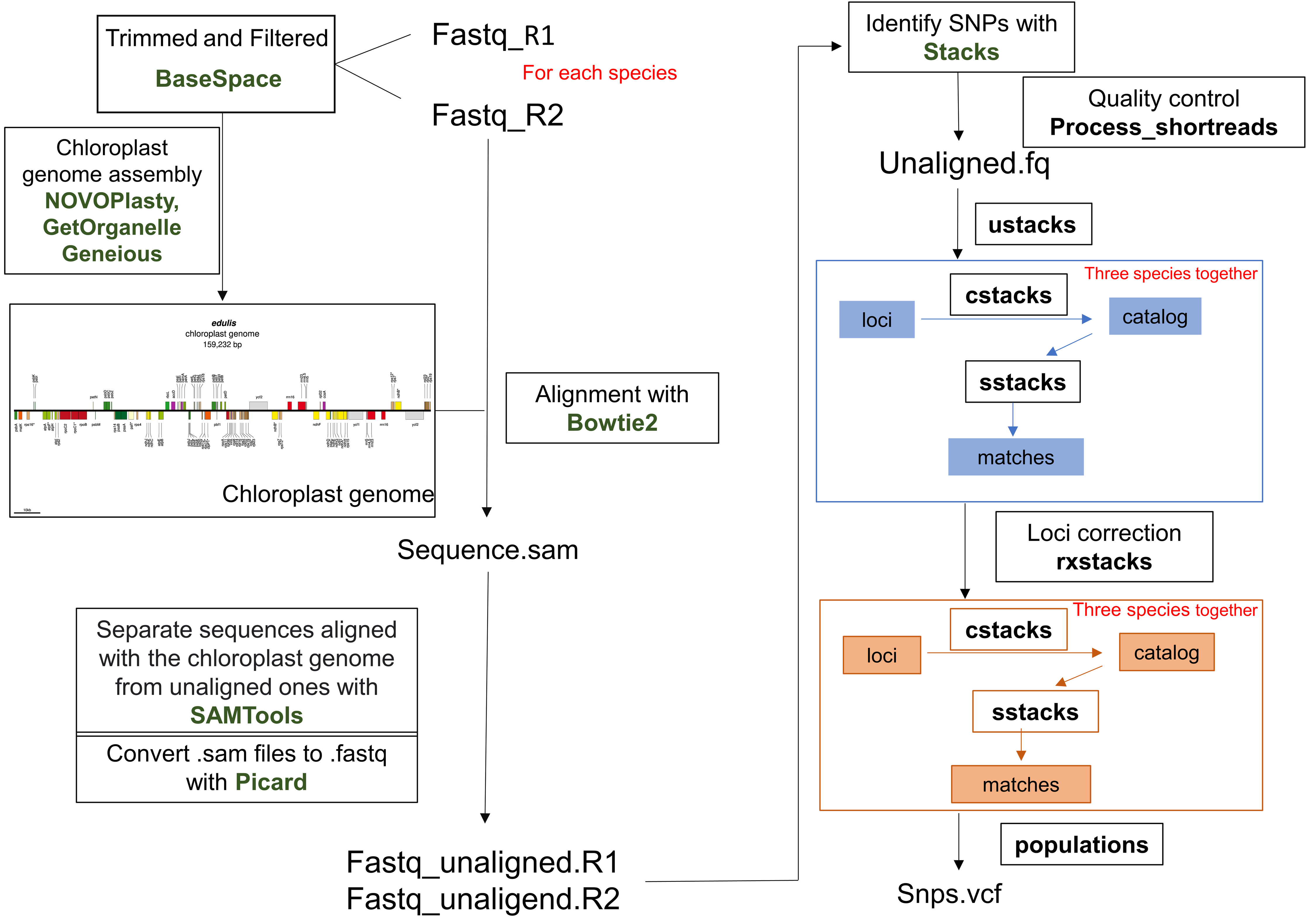

Supplement: S1 Fig — (TIF) [file pone.0266304.s001.tif]

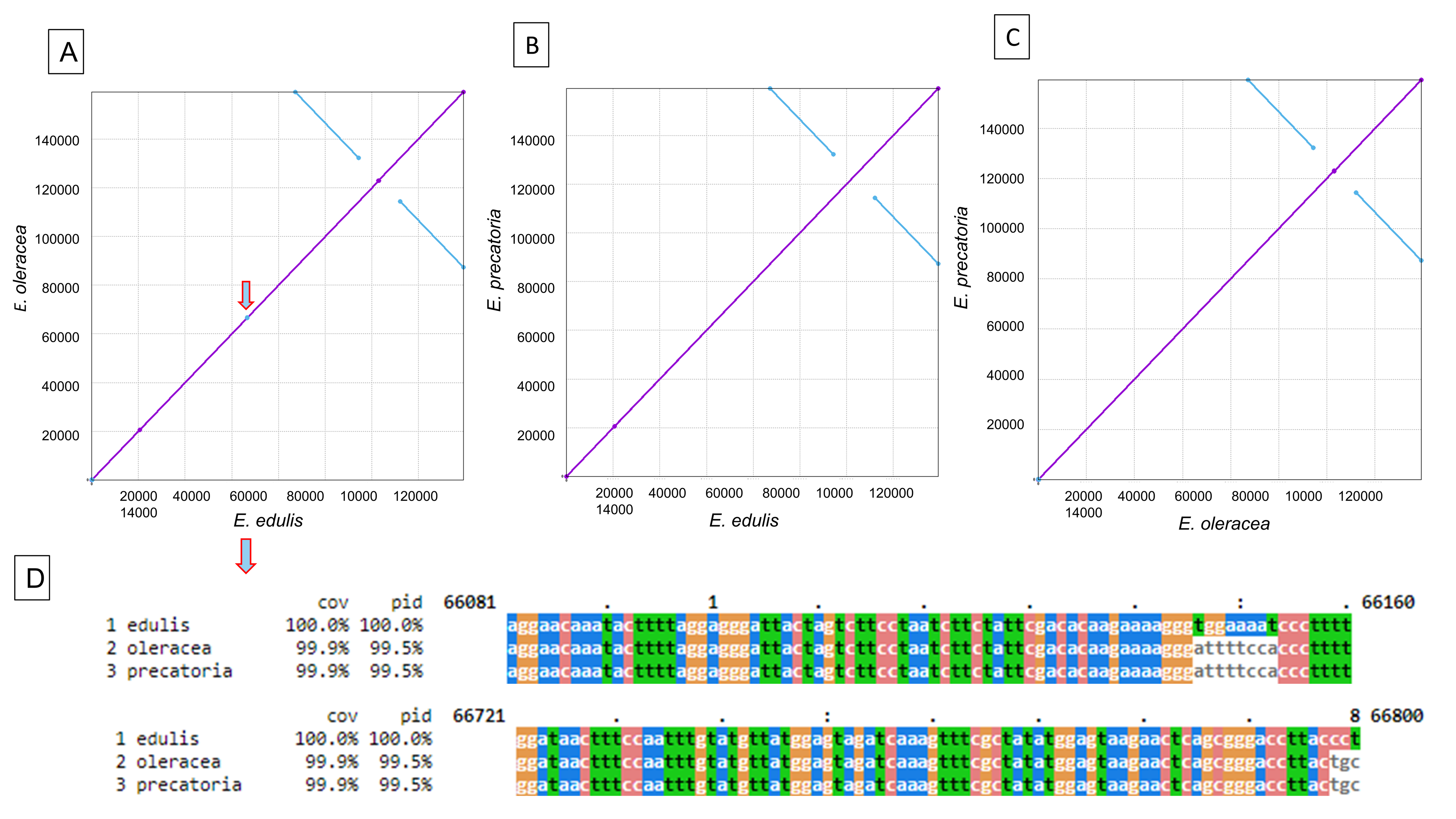

Supplement: S2 Fig — (A) Euterpe edulis (X–axis) x Euterpe Oleracea (Y-axis), (B) E. edulis (X-axis) x Euterpe precatoria (Y-axis), (C) E. oleracea (X–axis) x E. precatoria (Y-axis). The positive slope, in purple, represents the pair of sequences aligned and in the same orientation. The negative slope, in blue, represents the pair of sequence aligned, but in opposite orientation. The blue arrow in A highlights the region with inversions and SNPs between E. edulis and E. oleracea. (D) Local alignment with the chloroplast genomes of E. edulis, E. oleracea and E. precatoria in the region where inversions and SNPs were detected in A. (TIF) [file pone.0266304.s002.tif]

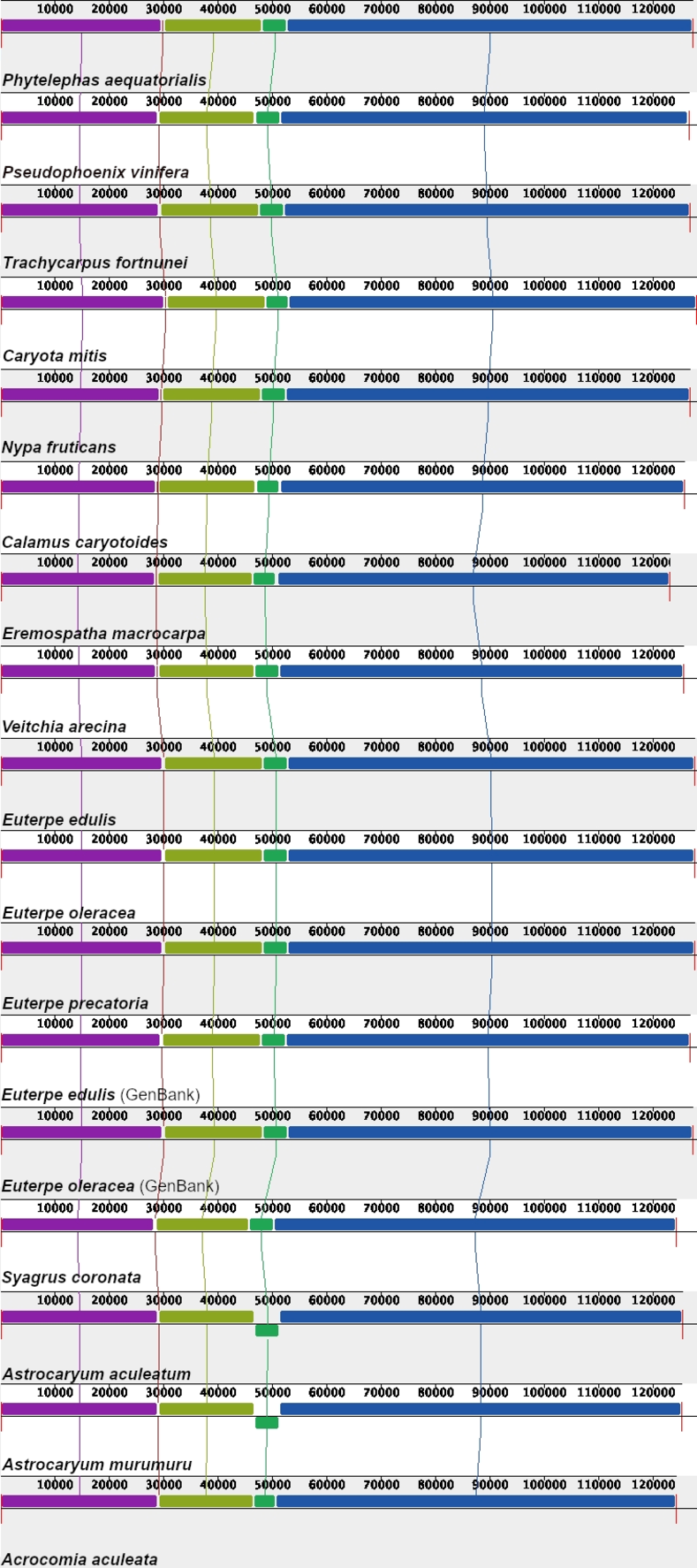

Supplement: S3 Fig — A sample of 20 different chloroplast genomes is shown. Color bars indicate syntenic blocks and the lines indicate the correspondence between them. Blocks on the top row are in the same orientation, while blocks on the bottom row are in inverse orientation. (TIF) [file pone.0266304.s003.tif]

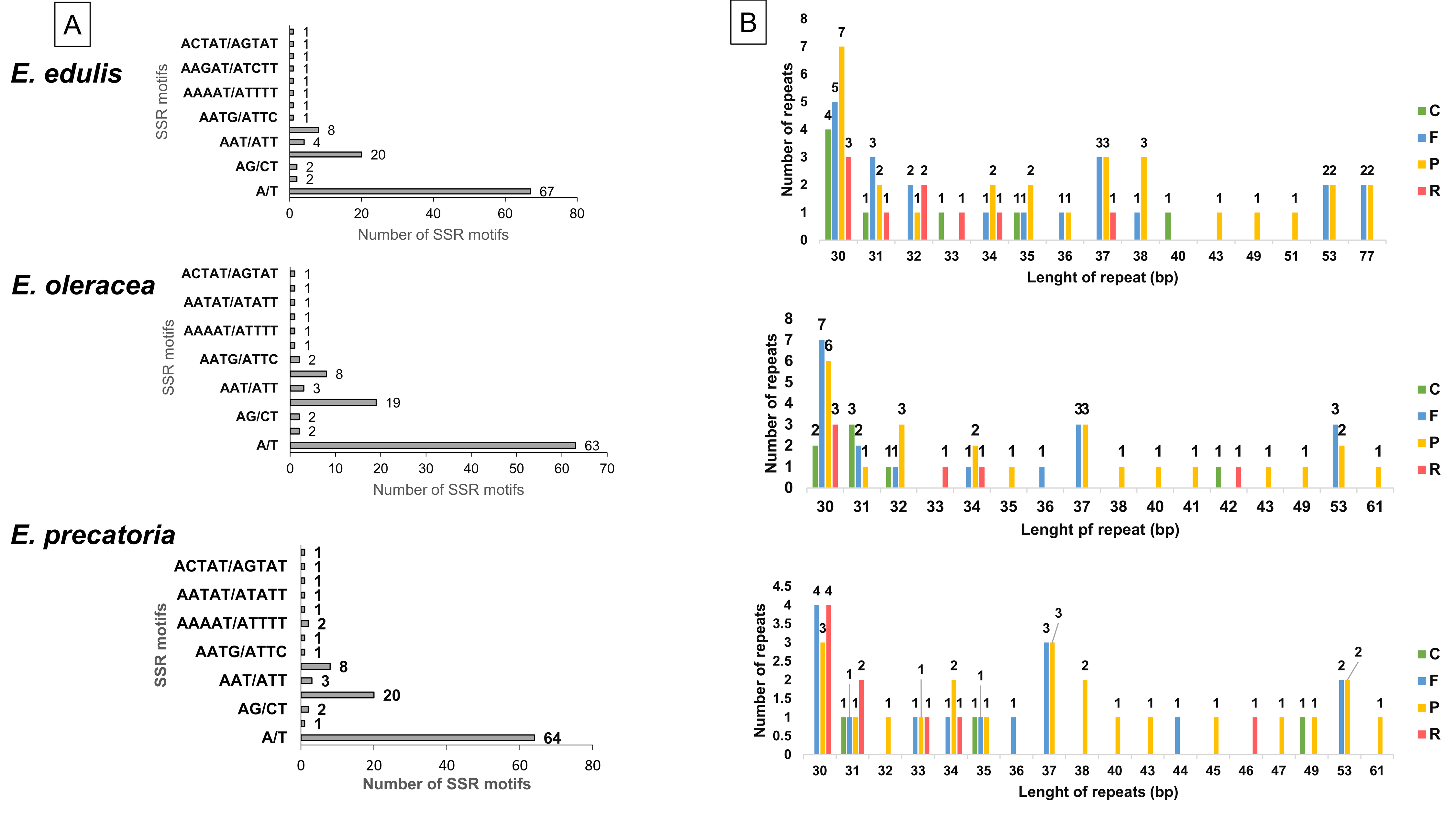

Supplement: S4 Fig — (A) The number of SSR motifs found in the three chloroplast genomes, considering sequence complementarities; (B) Number of dispersed repeats present in different size classes. (TIF) [file pone.0266304.s004.tif]

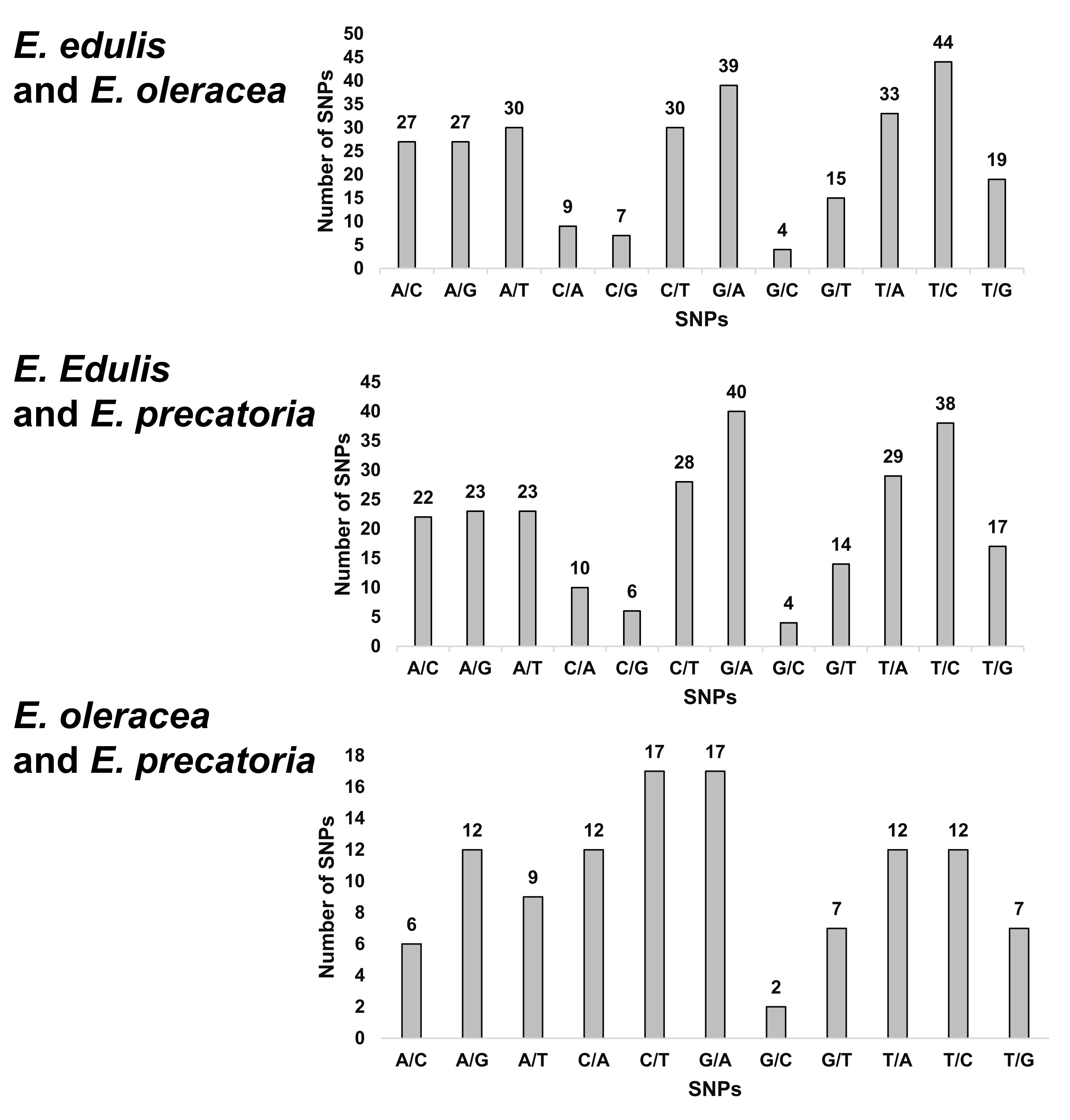

Supplement: S5 Fig — (TIF) [file pone.0266304.s005.tif]

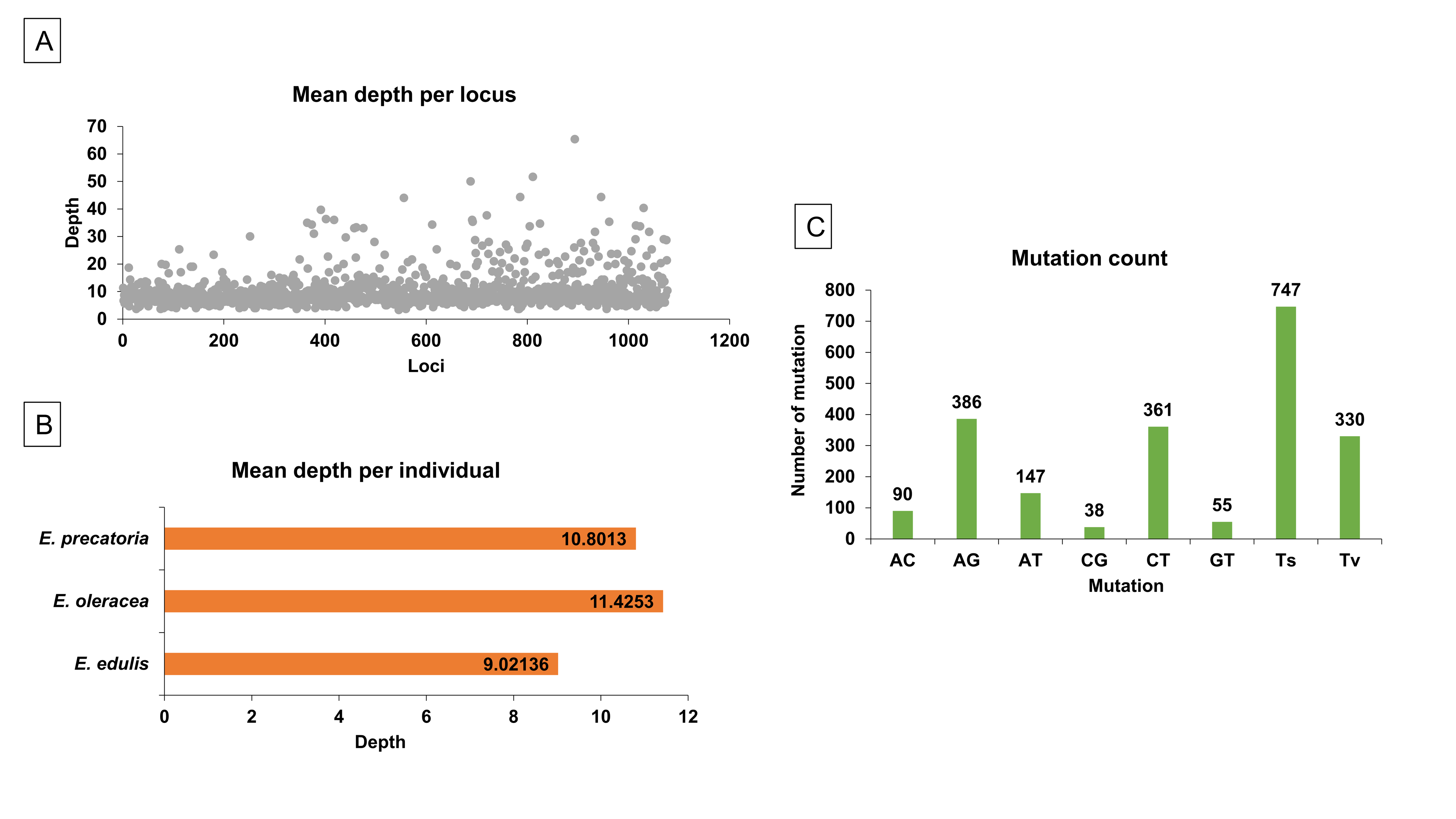

Supplement: S6 Fig — (A) Mean depth per loci; (B) Mean depth per species sequenced; (C) Mutation count in the present in the 1,077 SNPS. (TIF) [file pone.0266304.s006.tif]

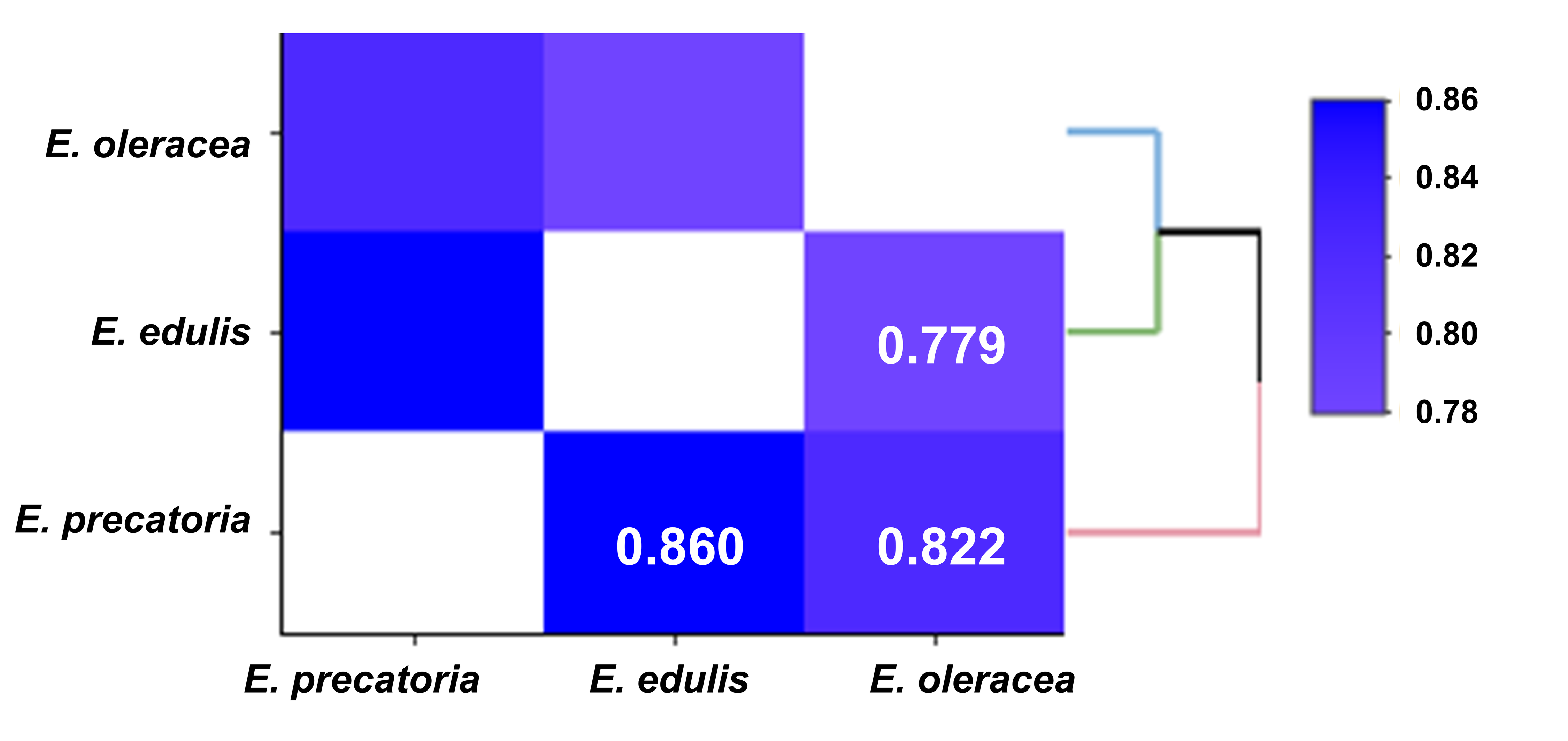

Supplement: S7 Fig — (TIF) [file pone.0266304.s007.tif]
